# Direct inhibition of c-Myc-Max heterodimers by celastrol and celastrol-inspired triterpenoids

**DOI:** 10.18632/oncotarget.6116

**Published:** 2015-10-14

**Authors:** Huabo Wang, Peter Teriete, Angela Hu, Dhanya Raveendra-Panickar, Kelsey Pendelton, John S. Lazo, Julie Eiseman, Toril Holien, Kristine Misund, Ganna Oliynyk, Marie Arsenian-Henriksson, Nicholas D. P Cosford, Anders Sundan, Edward V. Prochownik

**Affiliations:** ^1^ Section of Hematology/Oncology, Children's Hospital of Pittsburgh of UPMC, The University of Pittsburgh Medical Center, Pittsburgh, PA, USA; ^2^ Cell Death and Survival Networks Research Program, Sanford Burnham Prebys Medical Discovery Institute, La Jolla, CA, USA; ^3^ The Department of Pharmacology, The University of Virginia School of Medicine, Charlottesville, Virginia, USA; ^4^ The Department of Chemical Biology and Pharmacology, The University of Pittsburgh Medical Center, Pittsburgh, PA, USA; ^5^ The University of Pittsburgh Cancer Institute, Pittsburgh, PA, USA; ^6^ Department of Cancer Research and Molecular Medicine and The K. G. Jebsen Center for Myeloma Research, Norwegian University of Science and Technology, Trondheim, Norway; ^7^ Department of Microbiology and Tumor and Cell Biology, Karolinska Institutet, Stockholm, Sweden; ^8^ The Department of Microbiology and Molecular Genetics, The University of Pittsburgh Medical Center, Pittsburgh, PA, USA

**Keywords:** 10058-F4, 10074-G5, BET inhibitors, myeloma, neuroblastoma, quinone methide

## Abstract

Many oncogenic signals originate from abnormal protein-protein interactions that are potential targets for small molecule inhibitors. However, the therapeutic disruption of these interactions has proved elusive. We report here that the naturally-occurring triterpenoid celastrol is an inhibitor of the c-Myc (Myc) oncoprotein, which is over-expressed in many human cancers. Most Myc inhibitors prevent the association between Myc and its obligate heterodimerization partner Max via their respective bHLH-ZIP domains. In contrast, we show that celastrol binds to and alters the quaternary structure of the pre-formed dimer and abrogates its DNA binding. Celastrol contains a reactive quinone methide group that promiscuously forms Michael adducts with numerous target proteins and other free sulfhydryl-containing molecules. Interestingly, triterpenoid derivatives lacking the quinone methide showed enhanced specificity and potency against Myc. As with other Myc inhibitors, these analogs rapidly reduced the abundance of Myc protein and provoked a global energy crisis marked by ATP depletion, neutral lipid accumulation, AMP-activated protein kinase activation, cell cycle arrest and apoptosis. They also inhibited the proliferation of numerous established human cancer cell lines as well as primary myeloma explants that were otherwise resistant to JQ1, a potent indirect Myc inhibitor. N-Myc amplified neuroblastoma cells showed similar responses and, in additional, underwent neuronal differentiation. These studies indicate that certain pharmacologically undesirable properties of celastrol such as Michael adduct formation can be eliminated while increasing selectivity and potency toward Myc and N-Myc. This, together with their low *in vivo* toxicity, provides a strong rationale for pursuing the development of additional Myc-specific triterpenoid derivatives.

## INTRODUCTION

The pharmacologic deployment of small molecule inhibitors of transcription factors (TFs) is a major therapeutic goal for many diseases [1–4], particularly in cancer where aberrant TF expression is often a primary driver [5–8]. Although numerous strategies have been tested in pursuit of this objective, the most direct has been to identify chemical compounds that disrupt or prevent the TF's association with an obligate partner that is necessary for DNA binding and/or subsequent downstream oncogenic signaling [1–3, 8–12]. Unfortunately, the interacting surfaces of TFs and their partners are often quite large and devoid of topographic features that permit the easy design of specific and/or potent inhibitors [1, 9, 12, 13]. The interacting protein surfaces also frequently possess high free energies of association and/or exist in a state of intrinsic disorder (ID) prior to assembly. Small molecules may therefore be unable to disrupt pre-formed stable complexes and ID regions may lack structurally defined or stable binding sites [14–16]. Recently, however, new approaches have generated a number of promising candidate inhibitors that are addressing and gradually overcoming some of these barriers.

A therapeutically appealing oncogenic TF is c-Myc (Myc), which is frequently deregulated in transformed cells and is invariably required to maintain their high rates of proliferation, metabolism and ATP synthesis [17–21]. The inhibition of Myc promotes the regression of Myc-driven tumors as well as those not previously appreciated to be Myc-dependent [22–25]. Moreover, although Myc is also expressed by normal proliferating cells, its global inhibition in the whole animal is remarkably well-tolerated and associated with mild and reversible side-effects [22, 26, 27]. The potential utility of Myc inhibitors in a vast range of tumors, coupled with this low toxicity profile, provides further reason to continue the search for novel compounds.

We and others have identified small molecules that interfere with the interaction between and subsequent DNA binding by Myc and its obligate heterodimerization partner Max, which associate via homologous basic-helix-loop-helix-leucine zipper (bHLH-ZIP) domains [9, 12, 28–32]. Our characterization of some of these Myc inhibitors, most notably 10058-F4 and 10074-G5 and their analogs [28–30], has revealed that they interact specifically with and locally distort, short ID segments of contiguous amino acids within the bHLH-ZIP domain of Myc thus blocking its ability to dimerize with Max and bind DNA [33–35]. More recently, we showed that another small molecule termed JKY-2-169, deliberately engineered to interact with Myc in its Max-associated, α-helical conformation, causes a loss of DNA binding by distorting the heterodimer without dissociating it [31]. With the exception of small molecules that stabilize Max homodimers [36], it seems likely that most if not all other “direct” Myc inhibitors operate through one of these mechanisms [9, 12].

*In vivo,* direct Myc inhibitors demonstrate short half-lives, rapid metabolism and/or efflux from target cells and inefficient tumor penetration [30, 37, 38]. However, at least one direct Myc inhibitor, 10058-F4 [28], has demonstrated efficacy *in vivo* [26, 27]. In one case 10058-F4, which also binds to the bHLH-ZIP domain of the close Myc relative N-Myc [39], prolonged survival in an animal model of N-Myc-driven neuroblastoma [26]. In another case, nano-particle-mediated delivery of a 10058-F4 pro-drug increased the survival of mice bearing highly metastatic multiple myeloma xenografts [27]. These findings, together with recent improvements in the stability and cellular uptake of some of the original compounds [12, 29, 30] have provided encouragement that more tractable Myc (and N-Myc) inhibitors can be identified.

Given these considerations, we employed a yeast 2-hybrid-based approach to query a natural product library so as to identify more pharmaceutically amenable compounds [28]. This assay registered the triterpenoid celastrol as a moderately effective Myc inhibitor. Although celastrol and several related molecules inhibit a variety of tumor types both *in vitro* and *in vivo* [40–47], they often contain a highly reactive quinone methide moiety that promiscuously forms Michael adducts with free sulfhydryl groups [44, 48–50]. Not unexpectedly, numerous potential targets for celastrol have been identified and some, such as Hsp90, Cdc37, IKKB, and annexin II, are quite avid Michael adduct participants [48, 51, 52]. In fact, a previous mass spectroscopy-based survey showed that approximately 9% and 3% of proteins comprising the cellular and mitochondrial proteomes, respectively, are affected by celastrol [53]. Although many of these changes are likely secondary in nature and not attributable to Michael adduct formation *per se*, the large number of changes greatly diminishes the appeal of celastrol and related quinone methide-containing triterpenoids as viable chemotherapeutic agents.

Celastrol belongs to the family of triterpenoids that share a core structure of five fused carbocyclic rings. In contrast, many triterpenoids, including betulinic acid, oleanoic acid and ursolic acid lack the quinone methide moiety and instead possess a fully saturated core [41]. We show here that some triterpenoids lacking a quinone methide retain Myc inhibitory activity and in some cases are significantly better Myc inhibitors than celastrol itself. We further demonstrate their efficacy against several tumor types, particularly myelomas. Celastrol-inspired triterpenoids thus represent a new and intriguing class of agents that merits further consideration as potentially novel and more specific Myc inhibitors.

## RESULTS

### Identification of celastrol as a Myc inhibitor

**Figure 1:**
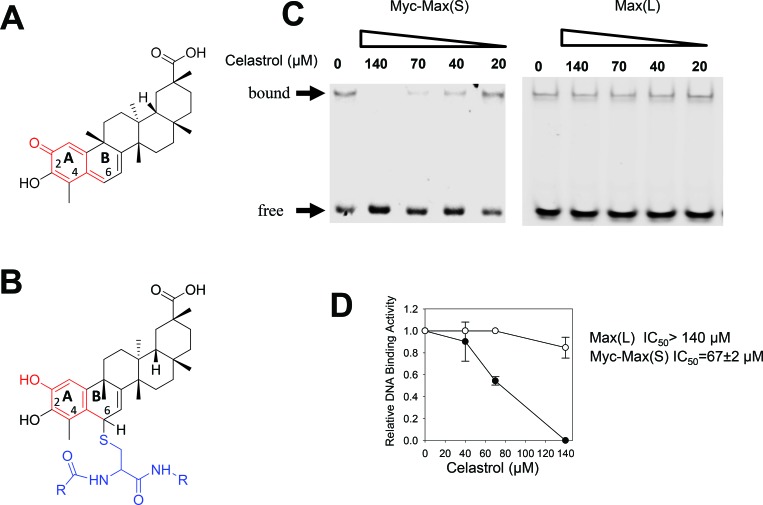
EMSA analysis of celastrol **A.** Celastrol structure. The portion of the molecule denoted in red shows the reactive quinone methide moiety. **B.** Formation of a typical Michael adduct with cysteine as an example (depicted in blue) is shown. Note that nucleophilic attack by free sulfhydryl-containing molecules occurs at position 6 of the B ring. **C.** Typical EMSA results in response to varying concentrations of celastrol. Recombinant Myc bHLH-ZIP domain (residues 353-437), and full-length Max(S) and Max(L) were used for EMSAs along with a HEX-tagged, double-stranded oligonucleotide containing a Myc binding E-box element (CACGTG) [30, 31]. Max(S) is the 151 residue isoform that homodimerizes efficiently but only binds DNA as a heterodimer in association with Myc [29, 30, 54, 55, 85]. The 160 residue isoform, termed Max(L), that does bind DNA as a homodimer and that closely mimics the Myc-Max(S) association was used as a control [54, 55]. **D.** Graphical representation of EMSA results from triplicate assays performed as described in C. IC_50_ values were defined as the concentration of celastrol required to interfere with half the binding of Myc-Max(S) heterodimers or Max(L) homodimers.

We used a yeast 2-hybrid assay to screen a library of naturally-occurring compounds for those that interfered with the Myc-Max-DNA interaction [28]. The most active compound identified was celastrol, a quinone methide triterpenoid originally isolated from a member of the Celastraceae family of creeping plants and known to practitioners of Chinese herbal medicine as the “Thunder of God” vine (Figure 1A) [44, 51, 52]. To confirm celastrol's effect on DNA binding by Myc, we employed electrophoretic mobility shift assays (EMSAs) using recombinant Myc and Max proteins [54, 55]. Notably, both proteins lacked free cysteines, thereby eliminating the possibility that any effects on DNA binding could be attributed to Michael adduct formation (Figure 1B). As seen in Figure 1C, celastrol prevented DNA binding by Myc-Max(S) heterodimers [56] with IC50s comparable to or lower than those for Myc inhibitors such as 10058-F4 and 10074-G5 [28] (Figure 1D). Celastrol was significantly less effective against Max(L) homodimers despite their weaker association and DNA binding [57] (Figure 1B and 1C). Thus, celastrol displays potency and specificity profiles comparable to those of other direct Myc inhibitors [12, 28–31, 58].

### Improved activity of select celastrol-inspired compounds lacking quinone methide moieties

Certain ester and amide derivatives of celastrol, including two termed CA16 and CA19, inhibit melanoma xenografts *in vivo* [41]. However, like celastrol, they contain a quinone methide moiety. On the other hand the structurally related triterpenoids betulinic, oleanoic and ursolic acids contain a saturated carbocyclic core structure. We therefore constructed a small library of analogs designed around these three acid scaffolds (Supplementary Figure 1) and tested them for their effects on DNA binding by Myc-Max(S) heterodimers. We also tested CA16 and CA19, as well as dihydrocelastrol-a reduced variant of celastrol-that contains an enone moiety.

Screening at high concentrations (100 μM) in the EMSA assay identified 7 active compounds (>50% inhibition-not shown). This, as well as greater specificity for Myc-Max(S) than for Max(L) was confirmed in subsequent concentration-response profiling with the most members (Supplementary Figure 2). Surprisingly, 3 compounds lacking quinone methides (SBI-0069272, SBI-0640599 and SBI-0640601-Supplementary Figure 1) were 4-6-fold more potent than celastrol (IC50s=10-15 μM) while continuing to demonstrate good specificity. A fourth analog, SBI-0061739, was somewhat less specific, with a ~2-fold preference for Myc-Max(S) heterodimers over Max(L) homodimers. Together, we refer to these as “SBI compounds”. The quinone methide moiety is therefore not only dispensable for the anti-Myc activity but in some cases is actually inhibitory.

**Figure 2:**
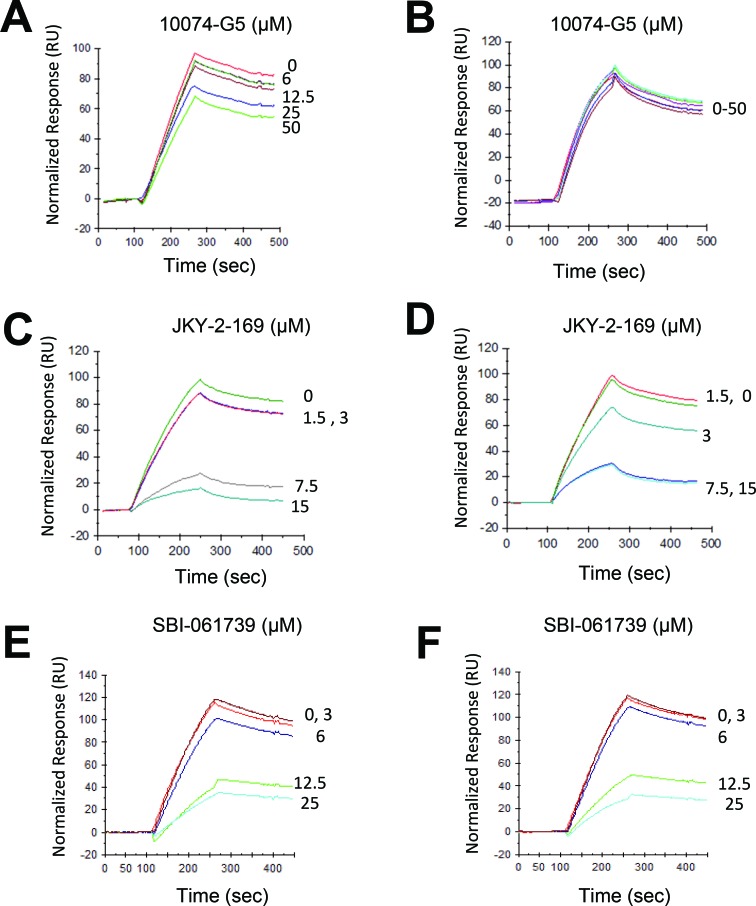
SPR analysis SBI-061739 abrogates DNA binding equally well when added prior to or following the formation of Myc-Max(S) heterodimers. **A.** A biotin-tagged E-box-containing double-stranded oligonucleotide was attached to a streptavadin-impregnated biosensor chip so as to achieve a response of 700-800 units as previously described [31, 59]. The reading was then re-set to 0. Purified recombinant Myc (20 nM) was allowed to interact with the indicated concentrations of 10074-G5, which binds to the Myc monomer [33, 34]. An equimolar amount of Max(S) was then added. Note the dose-dependent effect of 10058-F4 on Myc-Max(S) heterodimerization. **B.** The experiment described in A was repeated except that 10058-F4 was added to preformed Myc-Max(S) heterodimers. **C.**, **D.** The same experiments described in A and B were performed except that JKY-2-169 was added to the indicated final concentrations. **E.**, **F.** The same experiments performed with the indicated amounts of SBI-0061739. Neither the injection of individual Myc or Max(S) proteins nor any of the tested Myc inhibitors altered the response of the immobilized oligonucleotide (not shown).

To confirm on-target binding, we employed a surface plasmon resonance (SPR)-based approach [59] in which an E-box-containing oligonucleotide is tethered to the sensor chip of the SPR instrument and is followed by the addition of Myc and Max(S) in the presence of increasing amounts of inhibitor. Because compounds such as 10058-F4 and 10074-G5 prevent Myc-Max(S) heterodimerization by distorting Myc's bHLH-ZIP domain, they are most effective when added prior to heterodimerization (Figure 2A) [33, 34]. In contrast, their addition to pre-formed Myc-Max(S) heterodimers is much less effective due to the high free energy of association of the complex (Figure 2B) [57]. A much different pattern was observed with the α-helical mimetic JKY-2-169, which interferes with Myc-Max(S) DNA binding by distorting the heterodimer's conformation and is thus independent on the timing of its addition (Figure 2C and 2D) [31]. SBI-061739 was also equally effective irrespective of its addition relative to Myc-Max(S) heterodimerization thus suggesting that its mechanism of action closely mimics that of JKY-2-169 (Figure 2E and 2F). Similar but somewhat less pronounced effects were observed with celastrol (not shown).

### Celastrol distorts the structure of Myc-Max(S) heterodimers without causing their dissociation

^15^N-HSQC NMR spectroscopy has independently demonstrated JKY-2-169's mechanism of action [31]. Given that our SPR-based assessment showed that SBI-061739 altered Myc-Max(S) binding to DNA in a manner similar to that seen with JKY-2-169 (Figure 2), we explored further how celastrol itself affected the structure of Myc-Max(S). For this, we employed this same ^15^N-HSQC spectroscopy-based strategy [31], which allows the observation of interactions between small molecules and a protein of interest over a broad range of affinities. Even without residue-specific assignment, the technique provides insight into changes in secondary or overall structural arrangements and potentially allows the elucidation of affinities.

**Figure 3:**
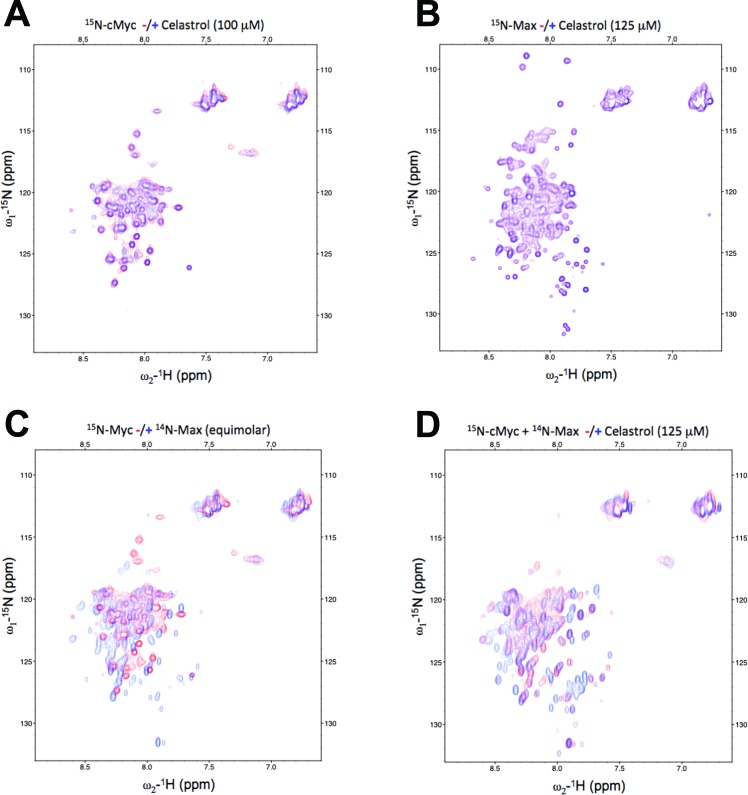
Celastrol alters the conformation only of the Myc-Max(S) heterodimer **A.** ^15^N-c-Myc HSQC spectra with (blue) and without (red) the addition of 125 μM celastrol. **B.** ^15^N-Max(S) HSQC spectra with (blue) and without (red) the addition of 125 μM celastrol. **C.** ^15^N-c-Myc only (red) and in heterodimeric association with Max(S) (blue). **D.** ^15^N-c-Myc/Max(S) binary complex alone (red) and in the presence of 125 μM of celastrol (blue).

To study how celastrol interacts with Myc and Max(S), ^15^N- or ^14^N-labeled recombinant proteins, purified as described for Figure 1, were used. At concentrations up to 100-125 μM, celastrol failed to provoke any observable chemical shift perturbations in Myc or Max(S), thereby indicating that it binds neither of the individual proteins (Figure 3A and 3B). We next formed the heterodimer by adding equimolar amounts of unlabeled Max(S) to ^15^N-Myc and observed the expected substantial change in the HSQC spectrum of ^15^N-Myc, indicative of increased α-helical content in the latter protein and reflecting its efficient heterodimerization (Figure 3C) [31, 57, 60]. The addition of celastrol to this heterodimer mediated additional, large chemical shift perturbations, indicating that the heterodimer is needed to provide a suitable binding site and that, following celastrol's addition, the final configuration attained is unlike that of either protein component alone (Figure 3D). These changes cannot be explained by non-specific effects such as protein aggregation as the line width of the resonances was unaffected over the entire celastrol concentration range (not shown). Similar studies performed with SBI-0640601 yielded equivalent spectral perturbations (not shown). Collectively, these data suggested that celastrol and SBI analogs, like JKY-2-169 [31], inhibits DNA binding by altering the Myc-Max(S) heterodimer's structure without causing its dissociation.

### Celastrol-inspired triterpenoids possess a cellular mechanism of action shared with other Myc inhibitors

In addition to direct Myc inhibitors such as 10058-F4, 10074-G5 and JKY-2-169, “indirect” Myc inhibitors have also been described [12]. These include JQ1, which inhibits the Myc-associated chromatin remodeling enzyme Brd4 and the synthetic lethal compound dihydroartemisinin, which activates the Ser/Thr kinase GSK3β that in turn phosphorylates and de-stabilizes Myc [12, 61]. Regardless of their class or structure, all these inhibitors promote cell cycle arrest, loss of Myc expression and the inhibition of Myc-regulated genes [21]. This is associated with a rapid depletion of cellular ATP stores that likely results from reduced glycolysis and oxidative phosphorylation, both of which are Myc-dependent [20, 62]. This leads to the up-regulation of AMP-activated protein kinase (AMPK), which works to restore ATP levels [63, 64]. Normally, this occurs by AMPK's suppression of proliferation and other energy-consuming processes until it can restore glycolysis and oxidative phosphorylation and replenish ATP supplies [63, 64]. In Myc's absence, however, these processes remain suppressed, thus leading to AMPK's chronic activation and the persistence of an ATP-depleted state [20, 62]. Recognizing their unmet energy demands, Myc-deficient cells respond by increasing fatty acid uptake and oxidation (FAO) and store the excess fatty acids as neutral lipid [26, 62]. In certain leukemia cells, Myc inhibitors also induce myeloid differentiation [21, 65, 66], which can be mimicked by depleting ATP without affecting Myc levels [21].

**Figure 4:**
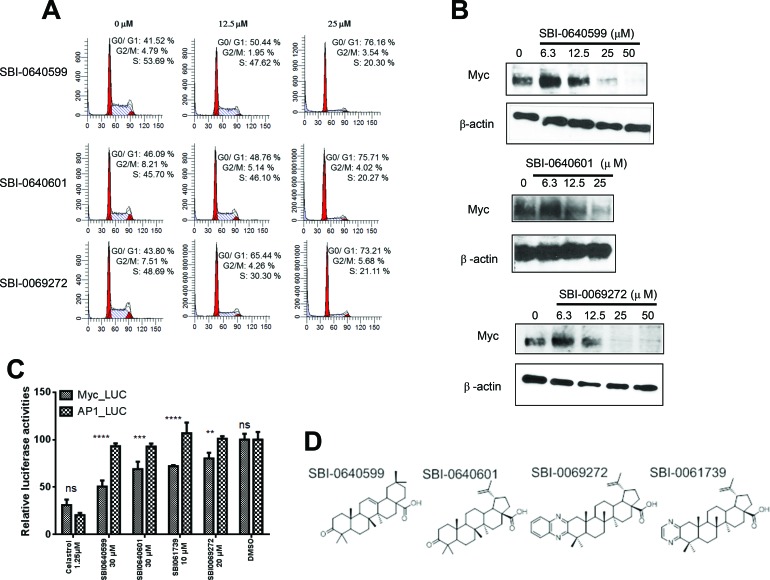
Celastrol and/or SBI compounds inhibit proliferation, promote Myc protein depletion and selectively inhibit a Myc-responsive promoter **A.** SBI compounds induce cell cycle arrest. HL60 cells in log-phase growth were exposed to the indicated concentrations of SBI compounds for 24-48 hr. Cells were then stained with propidium iodide and DNA content was quantified by flow cytometry [21, 30, 31]. **B.** SBI compounds promote Myc protein loss. HL60 cells in log-phase growth were exposed to the indicated concentrations of compounds for 24 hr. Total cellular lysates were then immuno-blotted for Myc protein. Staining of the same blots for β-actin was performed to serve as loading controls. **C.** Selective inhibition of a Myc-responsive promoter. HeLa cells stably expressing a highly labile luciferase under the control of either a Myc or AP-1-responsive promoter [59] were exposed for 4 hr to the indicated concentrations of celastrol or SBI compounds and then assayed for luciferase activity The results show the mean of triplicate samples +/− 1 S.E. ***: *p* < 0.05; ****: < 0.005 Immunoblotting for total Myc protein showed no changes compared to untreated control cells (not shown). **D.** Structures of the most relevant SBI compounds (also see Supplementary Figure 1).

**Figure 5:**
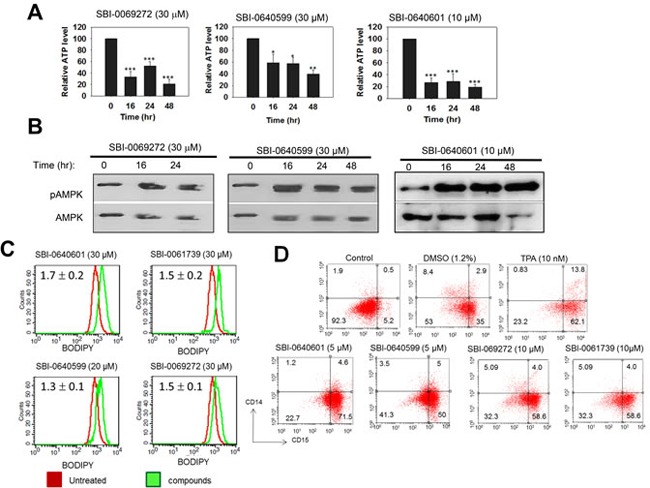
Myc inhibitors cause ATP depletion, activation of AMPK, accumulation of neutral lipid and promote myeloid differentiation **A.** SBI compounds were added to HL60 cells for the indicated periods of time. ATP levels were then quantified as previously described [62]. Shown are the values of quadruplicate determinations +/− 1 SE with the total ATP levels in untreated cells arbitrarily adjusted to 100%. p values were based on comparisons to untreated cells assayed in parallel. *: *p* < 0.05; **: *p* < 0.01; *** : *p* < 0.005. **B.** SBI compounds activate AMPK. Analogs were added to log-phase HL60 cells for the indicated periods of time. The cells were then evaluated by immuno-blotting for total AMPK or for the Thr_172_-phosphorylated, active form of the enzyme (pAMPK). **C.** SBI compound-treated cells accumulate neutral lipid. HL60 cells were exposed to the indicated compounds for 3 days and then stained for 30 min with BODIPY-493/503, which was quantified by flow cytometry [62]. Numbers in the upper left corner indicate the mean relative ratio of fluorescence intensities between analog-treated cells (red curves) and vehicle-treated cells (green curves) in three independent experiments +/− 1 standard error. **D.** Induction of myeloid differentiation by SBI compounds. HL60 cells were incubated with the indicated agents for 4-5 days. Cells were then immuno-stained to detect the expression of CD14 (myeloid) and CD15 (monocyte/macrophage) cell surface antigens [21]. DMSO- and 12-O-tetradecanoylphorbol-13-acetate (TPA)-treated cells served as controls for “pure” myeloid and monocyte/macrophage differentiation, respectively [67, 68]. Cell surface fluorescence was quantified by two-color flow cytometry [21].

All the above processes were faithfully recapitulated in HL60 human promyelocytic leukemia cells in which SBI analogs induced a concentration-dependent cell cycle arrest that correlated with a decline in intracellular Myc protein levels (Figure 4A and 4B). Thus, within 4 hr of their addition, SBI analogs selectively inhibited the expression of a Myc-responsive promoter relative to that of an AP-1-responsive promoter whereas celastrol itself was somewhat more selective for the latter (Figure 4C and 4D). These results are further consistent with our finding that the absence of an active quinone methide confers greater Myc specificity to the resultant compounds. Additional findings included a marked depletion of ATP and the phosphorylation-dependent activation of AMPK (Figure 5A and 5C). Staining with the neutral lipid-specific dye BODIPY-493/503 was also enhanced (Figure 5C) and was consistent with prior observations in other cell types following Myc or N-Myc inhibition [26, 62]. Finally, cells up-regulated the myeloid cell surface antigen CD15 at levels comparable to those seen in response to other Myc inhibitors or the classical myeloid differentiating agent DMSO (Figure 5D) [67]. In contrast, treatment with the phorbol ester 12-O-tetradecanoylphorbol-13-acetate (TPA) induced a more myelo-monocytic differentiation as evidenced by the concurrent expression of the CD14 cell surface antigen [68]. Together with the results displayed in Figure 4, these findings show that, like other Myc inhibitors, SBI analogs promote cell cycle arrest, de-stabilize Myc protein, deplete cellular ATP stores and activate AMPK. Their induction of myeloid maturation is also consistent with Myc's known role in suppressing differentiation [69–72].

### SBI compounds inhibit the proliferation of multiple cancer types

Celastrol and some related triterpenoids inhibit numerous cancer types, both *in vitro* and *in vivo* [41–46]. We therefore tested the above SBI compounds for *in vitro* activity against several cancer types. Initially, these included HL60 and Burkitt lymphoma cells because they express extremely high levels of Myc and respond to Myc inhibitors by undergoing proliferative arrest and apoptosis [73] (Figures 4 and 5). As seen in Table 1, celastrol and the SBI analogs tested inhibited the proliferation of both cell types. Unsurprisingly, celastrol was the most potent compound, despite being the least effective Myc inhibitor (Figure 1C).

**Table 1:** Inhibition of Daudi Burkitt lymphoma and HL60 promyelocytic leukemia cell lines by celastrol and select triterpenoids

IC50 (μM):							
	Celastrol	CA16	CA19	SBI-0640599	SBI-0640601	SBI-0069272	SBI-0061739
HL60	0.46 ± 0.06	34.77 ± 4.07	19.36 ± 2.76	11.98 ± 1.28	13.24 ± 1.74	10.77 ± 2.18	0.82 ± 0.23
Daudi	0.09 ± 0.02	13.82 ± 1.42	5.92 ± 0.97	14.25 ± 3.7	6.36 ± 1.25	14.26 ± 3.2	3.19 ± 2.28

**Figure 6:**
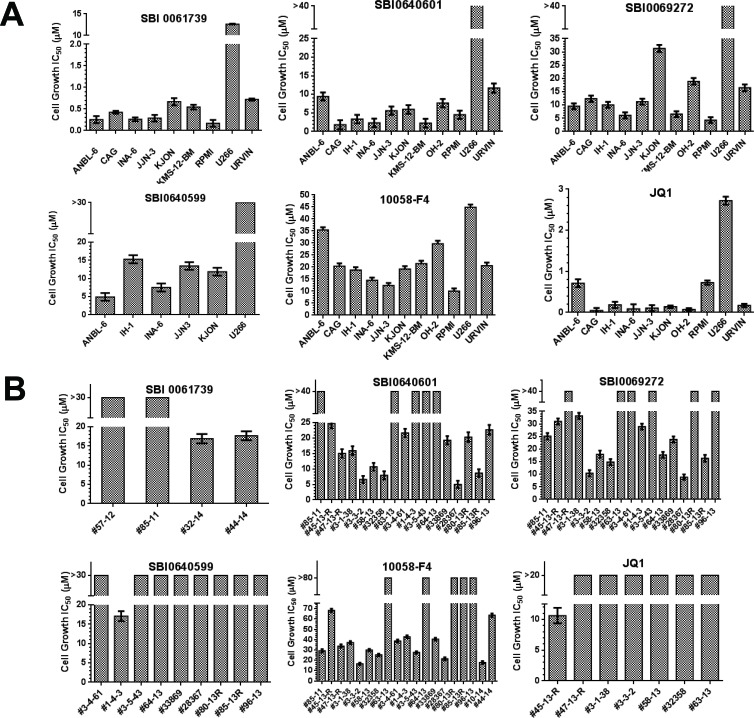
Susceptibilities of multiple myeloma cell lines and primary explants to SBI compounds **A.** Cell lines. The indicated human myeloma cell lines were treated with the indicated compounds for 3 days at which point the total viable cell count was determined using the Cell Titer Glo assay method. Shown are the IC50 values that were calculated from standard 10-point dose-response curves (Supplementary Figure 3). **B.** Primary myeloma explants. Myeloma cells were purified from bone marrow aspirates [76] and maintained in culture for 3 days in the presence of varying concentrations of the indicated compounds [86] IC50 values were calculated from dose-response curves (Supplementary Figure 4).

We next examined multiple myeloma (MM) cell lines given that many are associated with de-regulated Myc expression and are susceptible to some Myc inhibitors [17, 27, 74–76]. 10058-F4 and JQ1, chosen as representative members of the direct and indirect classes of inhibitors, respectively [12], inhibited nearly all cell lines tested, with JQ1 being more effective in all cases by a factor of nearly 90 (mean IC50: 0.25 μM *vs*. 21.8 μM) (Figure 6A and Supplementary Figure 3). SBI analogs showed intermediate activities ranging from 0.26-67.3 μM with SBI-0061739 being the most potent. Indeed, 8 of 9 cells lines showed IC50 values of <1 μM for this compound. Interestingly, the most resistant myeloma line tested, U266, is the only one that expresses L-Myc instead of Myc [76]. Disregarding this cell line, the mean IC50 for JQ1 (0.25 μM) was <2-fold lower than that for SBI-0061739 (0.42 μM).

With only a single exception, primary myeloma cells were resistant to JQ1 whereas the majority were sensitive to 10058-F4 in a concentration range similar to that observed for established myeloma cell lines (Figure 6A and Supplementary Figure 4). Notably, most myelomas were even more sensitive to SBI compounds (SBI-0069272, SBI-0640601 and SBI-0061739) than to 10058-F4 and JQ1.

Given the apparent superiority of SBI-0061739 against myelomas, we conducted a broad screen with this compound against the National Cancer Institute NCI-60 cell line collection. Overall, 17 cell lines (28%) were highly sensitive to SBI-0061739, with IC50 values <2 μM (Supplementary Figure 4). Although the numbers of cell lines for any specific cancer were relatively small, particularly sensitive types included leukemias (4/6 lines), non-small cell lung cancer (3/9 lines) and breast cancer (3/7 lines). Ovarian and renal cancers appeared the most resistant, with all 6 lines from the first group and 7 of 8 cell lines from the second group showing IC_50_ values >2 μM. Thus, myelomas are particularly sensitive to SBI compounds and specifically to SBI-0061739.

**Figure 7:**
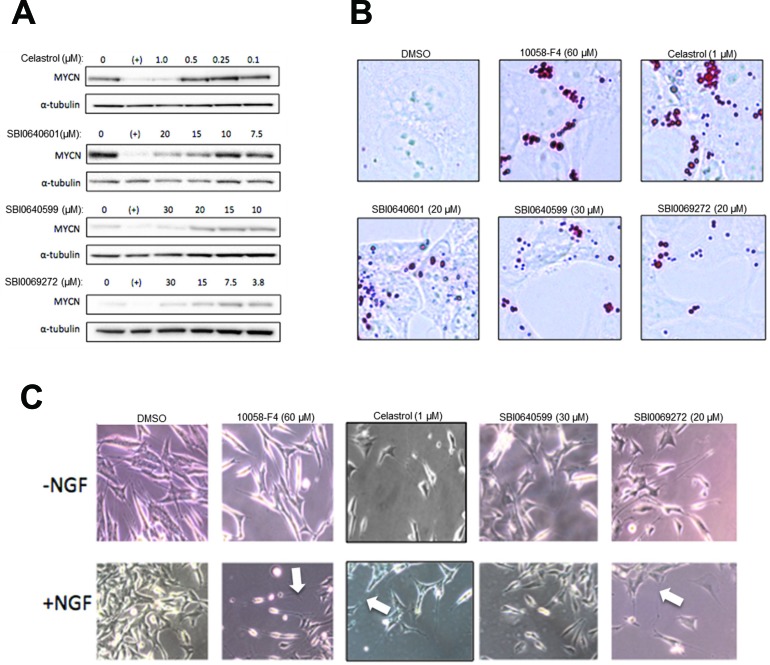
N-Myc-amplified neuroblastoma cells are targets for celastrol and SBI analogs **A.** SK-N-BE(2) cells were exposed to celastrol or the indicated SBI compounds for 24 hr prior to immunoblotting for N-Myc protein. As positive and negative controls, respectively, parallel cultures were exposed to 60 μM 10058-F4 (+) or to DMSO vehicle only. **B.** SK-N-BE(2) cells treated as described in A were fixed and stained with Oil Red O to delineate lipid droplets. **C.** SK-N-BE(2) cells were exposed to the indicated amounts of Myc inhibitors for 5 days either in the concurrent absence or presence of nerve growth factor (NGF: 50 ng/ml). Typical fields of unfixed cells were then photographed. Note the presence of longer and more numerous neurite outgrowths in cells exposed to Myc inhibitors (arrows).

Neuroblastoma cells respond to previously-described Myc inhibitors by down-regulating N-Myc, undergoing proliferative arrest, terminally differentiating and accumulating neutral lipid droplets [26]. The effects of celastrol and SBI analogs SBI-0640599 and SBI-0069272 on N-Myc-amplified SK-N-BE(2) human neuroblastoma cells were similar. As seen in Figure 7A, N-Myc protein declined within 24 hr of adding 10058-F4, celastrol or SBI compounds. The cells also accumulated neutral lipid (Figure 7B) and eventually differentiated as evidenced by an increase in the number and length of neurite protrusions, which were further enhanced by nerve growth factor (Figure 7C). Celastrol and certain SBI compounds thus closely phenocopy 10058-F4 [26].

## DISCUSSION

While celastrol and several of its analogs possess significant anti-neoplastic effects *in vitro* and *in vivo* [40, 41, 43, 45, 47, 52, 53, 65, 77, 78], their clinical implementation has been hampered by their chemical promiscuity [45, 46, 48, 50, 52, 53, 65, 77, 78]. Therefore the discovery of compounds that replicate the therapeutic benefits of celastrol, while minimizing its non-specific properties, would represent a key advancement.

By synthesizing and testing specific triterpenoid derivatives, we found certain celastrol-inspired compounds lacking the quinone methide moiety to be significantly better than celastrol at disrupting Myc-Max(S) DNA binding. Further evidence that the quinone methide group's negative effect on DNA binding was unrelated to any aspect of Michael adduct formation was the fact that none of the recombinant Myc or Max proteins used in the current work contained cysteine residues. Thus, the more efficient interaction of SBI compounds with Myc-Max(S) heterodimers argues that the quinone methide group is not only dispensable for the anti-Myc effect but is actually detrimental. The future design of triterpenoids lacking this group will likely address how to further increase both potency and specificity.

The absence of a quinone methide moiety in all four of the most potent SBI compounds, while significantly increasing their Myc specificity, concurrently decreased their anti-proliferative efficacy against cancer cell lines (Table 1, Figure 6 and unpublished data). This was not unexpected given that a number of celastrol's most prominent targets participate in Michael adduct formation [45, 46, 48, 50, 52, 53, 65, 77]. For example, the potent inhibitory effect against NF-kB, is at least partly mediated by celastrol's adduct formation with the regulatory protein IKKB [49]. Celastrol's potent inhibition of Hsp90 is also likely indirect and mediated by the formation of a disulfide linkage with the Hsp90 partner p37 [79]. The observation that free thiols block many of celastrol's biological effects [50] suggests that they are mediated by numerous other promiscuous disulfide bond-forming reactions. These results imply that eliminating the quinone methide function of celastrol should reduce its overall potency by virtue of minimizing its promiscuous interactions while improving its inhibition of Myc.

As was true for other Myc inhibitors, cell lines showed different susceptibilities to SBI analogs (Figure 6 and Supplementary Figure 4). This was perhaps best illustrated with myelomas. Discounting the previously discussed U266 line, which neither expresses Myc nor is sensitive to 10058-F4 [76], we observed as much as 3-4-fold differential susceptibilities in response to 10058-F4, 20-fold differences in response to the bromodomain inhibitor JQ1 and up to 6-fold differences in response to SBI compounds. JQ1 was usually the most potent compound, which is consistent with its being a highly effective, albeit indirect, Myc inhibitor [80–83].

Several major differences were noted between the above cell lines and primary myeloma explants however. First, JQ1, was virtually without effect against primary myelomas, even at concentrations exceeding 10-20 μM (Figure 6B). Second, primary explants sensitive to 10058-F4, were usually sensitive to at least one, if not all, of the tested SBI compounds. Conversely, primary myelomas resistant to 10058-F4 also tended to be resistant to SBI compounds. This suggests that, at least in the examples presented here, the relative sensitivity of a primary myeloma is determined more by intrinsic properties of the tumor rather than by the nature of the Myc inhibitor. While finding reasonable correlations between Myc expression and sensitivity to 10058-F4, we were unable to show these in primary myelomas. It will clearly be important in future work to determine the basis for these observed differential therapeutic susceptibilities.

It was not surprising that the effects of celastrol and SBI compounds on neuroblastoma cells were similar to those seen with 10058-F4 (Figure 7). This is in keeping with the close functional relationship between Myc and N-Myc [26, 39] and supports the idea that, as a group, Myc inhibitors may find application in the treatment of neuroblastoma where N-Myc over-expression is highly correlated with disease stage and survival [84].

Celastrol and some of its quinone methide-containing analogs are active against a range of tumors and are surprisingly well-tolerated *in vivo* [40, 41, 43, 45–47, 70, 78]. These favorable pharmacologic profiles suggested that quinone methide-lacking analogs with reduced target promiscuity might be even better tolerated but also less potent due to their more focused biological effects. Indeed, our preliminary studies support this by showing that the most potent SBI analogs tested *in vivo* are tolerated at i.v. doses as high as 20-40 mg/kg/day (not shown). As predicted however, they are less potent than other celastrol analogs with intact quinone methide moieties such as CA19 [41] and we have yet to demonstrate sustained effects against murine models of human myeloma xenografts (not shown). Future attempts to optimize Myc-targeted triterpenoids therapeutically may require not only improving potency against Myc itself but also perhaps on restoring at least partial off target activity.

## MATERIALS AND METHODS

### Natural product library screening

A yeast two hybrid system that allows the Myc-Max interaction to be quantified was screened as described previously [28] with a 720 member collection of natural products that includes complex oxygen heterocycles, sesquiterpenes, diterpenes alkaloids, sterols, pentacyclic triterpenes, and other compounds (Discovery Systems, Inc. Gaylordsville, CT). All compounds were supplied as 10 mM stocks in DMSO and were screened in duplicate wells at 10 μM concentrations. Compounds registering as “hits” were screened again at varying concentrations for verification before being advanced for further study.

### Chemical synthesis of triterpenoids

Celastrol, betulinic acid, oleanolic acid and ursolic acid were purchased from commercial sources and derivatized according to previously reported procedures [44, 75]. Details for the synthesis of the SBI analogues, including synthetic schemes, experimental procedures and spectral characterization data are available in Supplementary Materials and Methods. CA 16 and CA 19 were synthesized according to published procedures [41, 78].

### Purification of recombinant Myc and Max proteins and EMSA assays

The 83 residue His_6_-tagged Myc bHLH-ZIP domain (amino acids 354-437), and full-length His_6_-tagged Max(S) and His_6_-tagged Max(L) were expressed and purified as previously described [30, 85]. 20 nmol each of Myc and Max(S) or 40 ng of Max(L) were used for EMSA assays that included 30 nmol of a (HEX)-tagged double-stranded E-box-containing oligonucleotide (IDT, Coralville, IA, USA) [30, 85]. Importantly, the Myc protein excluded the two C-terminal amino acids (Cys_438_ and Ala_439_) from the final sequence, thus eliminating a potential reaction site for quinone methide-containing triterpenoids.

### Cell culture and viability assays

Cell lines were propagated in RPMI medium or Dulbecco's modified Eagle's minimal essential medium (D-MEM), containing 10% fetal bovine serum, 100 units/mL penicillin G and 100 μg/mL streptomycin. Proliferation and viability assays, cells were performed in 96 well plates in the presence of serial dilutions of compounds. Relative cell numbers and viability were determined 2-4 days later using MTT or CellTiter Glo (Promega, Inc. Madison, WI) assays as previously described [29, 30, 76, 86].

Primary myeloma samples were obtained from the Norwegian Myeloma Biobank after obtaining approval from the Regional Ethics Committee.

### Surface plasmon resonance (SPR) and NMR spectroscopy studies

SPR experiments were performed as described previously [59] using a Biacore™ 3000 instrument and streptavidin-coated biosensor chips (SA-Chip, GE Healthcare, Inc. Piscataway, NJ). The running buffer used was HBS-EP (10mM HEPES, pH 7.4; 500 mM NaCl; 3 mM EDTA; 0.005% v/v Surfactant P20) [59]. The E-box-containing oligonucleotide was attached over a 30 min period at a rate 10 μL/min and adjusted to achieve binding levels of ~700-800 RU, which was then reset to 0 prior to the application of proteins.

The measurement of Myc-Max(S) binding to the oligonucleotide and its disruption by inhibitors were performed in HBS-EP buffer at a flow rate of 60 μL/min to minimize any mass-transport limitation effects. Protein solutions, containing 20 nM each of Myc and Max(S), were injected for 300 seconds. Protein-DNA complexes were dissociated in HBS-EP buffer for 100 seconds. Individually, neither Myc monomer nor any Myc inhibitors altered the response of the immobilized oligonucleotide (not shown).

NMR Experiments were performed on a Bruker Avance 600 MHz spectrometer (Billerica, MA). A standard ^1^H/^15^N fast HSQC pulse sequence was used with the Max/Myc proteins at 298K. The chemical shifts were referenced to the ^1^H_2_O signal at 4.87 ppm. Data were processed using NMRPipe (http://spin.niddk.nih.gov/NMRPipe/). Spectra were analyzed using a modified version of Sparky (https://www.cgl.ucsf.edu/home/sparky/manual/files.html).

### Cell cycle analyses, flow cytometry, immuno-blotting and ATP assays

Cell cycle analyses were performed as previously described [21, 29, 30]. Briefly, test compounds were added to logarithmically-growing cells for 24-48 h. Cells were then washed twice in PBS and stained with propidium iodide. Cell cycle profiles were generated using a BD FACSCalibur flow cytometer (Becton Dickinson, Inc. Franklin Lakes, NJ, USA) and analyzed with ModFit LT 3.3 software (Verity Software House, Topsham, ME, USA).

To monitor HL60 differentiation, we quantified the expression of macrophage/monocyte-specific cell surface antigen CD14 and myeloid-specific cell surface antigen CD15 using fluorescently-tagged monoclonal antibodies (mAbs) as previously described [21]. Two-color flow cytometry was performed on a BD FACSCalibur™ flow cytometer and analyzed using CellQuest Pro software (BD Biosciences) and Flowing Software 2.5.1 (www.flowingsoftware.com).

Immunoblotting was performed as previously described [21, 29, 30]. Antibodies included a mouse mAb against human Myc (1:500 dilution: Clone 9E10, Santa Cruz Biotechnology, Inc. Santa Cruz, CA), a mouse mAb against β−actin (1:10,000 dilution: cat. no.3700S Cell Signaling Technology, Inc. Beverly, MA), a mouse mAb against AMPK (1:1000 dilution: catalog no. 2532, Cell Signaling Technology) and a rabbit polyclonal antibody against AMPK phospho-Thr_172_ (1:1000 dilution, catalog no.2535, Cell Signaling Technologies).

For ATP assays, cells were exposed to test compounds in 12 well plates for 24-18 h and were then counted and lysed in 96 well plates [20, 21, 62]. ATP was quantified in quadruplicate with an ATP Lite ATP detection kit (Perkin-Elmer, Inc. Downers Grove, IL). Luminescence was adjusted to the input cell number and expressed as a relative value compared with an untreated control. Statistical analyses utilized GraphPad Prism 5 Software (GraphPad Software, Inc. La Jolla, CA). P values were determined with Dunnett's Multiple Comparison Test.

### Luciferase reporter assays

HeLa cells were stably transfected with pGL4.28luc2CP/minP/Hygro plasmids (Promega, Madison, WI, USA) encoding a highly unstable luciferase expression cassette under the control of minimal promoter, upstream of which were included three tandemly repeated Myc or AP-1 binding sites [21]. Luciferase assays were performed on triplicate wells of logarithmically growing cells exposed for 6 h to the compounds of interest. Cells were harvested, washed twice and re-suspended in 70 μL of cell lysis buffer (One-Glo Luciferase Assay System Kit, s), 50 μL of which was assayed using the conditions recommended by the provider.

## SUPPLEMENTARY MATERIAL FIGURES


