# Supplementary material for: Direct inhibition of c-Myc-Max heterodimers by celastrol and celastrol-inspired triterpenoids
Source: Oncotarget. 2015 Oct 14;6(32):32380–95. doi: 10.18632/oncotarget.6116 (PMC4741700; doi:10.18632/oncotarget.6116)
Supplement: Supplementary file 1 [file oncotarget-06-32380-s001.pdf]

# Direct inhibition of c-Myc-Max heterodimers by celastrol and celastrol-inspired triterpenoids

## Supplementary Material

### Chemistry: Methods, Experimental Procedures and Characterization

**General**

All reagents were obtained commercially and were used without purification. Reactions were performed with magnetic stirring in oven-dried glassware under an inert atmosphere (argon). Chromatography was performed with an Isco Companion system using pre-packed RediSep silica cartridges (Teledyne Isco, Inc. Lincoln, NE). $^{1}$H- and $^{13}$C-NMR spectra were obtained on a Jeol 400 spectrometer (Jeol USA, Inc., Peabody, MA) at 400 MHz and 100 MHz, respectively. Chemical shifts are reported in $\delta$ (ppm) relative to residual solvent peaks or TMS as internal standards. Coupling constants are reported in Hz. LC-MS analyses were performed on a Shimadzu 2010EV LCMS (Sapporo, Japan) using the following conditions: Kromisil C18 column (Sigma-Aldrich) (reverse phase, 4.6 mm × 50 mm); a linear gradient from 10% acetonitrile and 90% water to 95% acetonitrile and 5% water over 4.5 min; flow rate of 1 mL/min; UV photodiode array detection from 200 to 300 nm. High-resolution ESI-TOF mass spectra were acquired by the Mass Spectrometry Core at Sanford Burnham Prebys Medical Discovery Institute (Orlando, Florida). Purity was determined to be >98% for all tested compounds.

**SBI-0640601**[1]

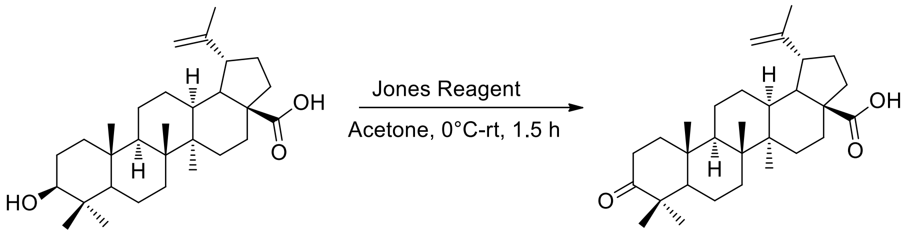


**General Method A**: To a solution of Betulinic acid (1 g) in acetone (100 mL) at 0º C, was added freshly prepared Jones reagent in drop-wise until the reaction mixture retained the yellowish-orange color of the reagent. It was then gradually warmed to room temperature and stirred for additional 1.5 h. Excess reagent

was quenched by the addition of methanol, and stirred for an additional 15 min. Excess water was added to the reaction mixture, the precipitate was collected by filtration, washed with water and dried. The crude product was purified by column chromatography using hexanes:ethyl acetate solvent system (100% hexanes to 10% EtOAc in Hexanes). White solid (0.900 g, 90%). $^{1}$H NMR ($CDCl_3$): $\delta$ 4.73 (d, $J$ = 1.8 Hz, 1H), 4.61 (s, 1H), 3.04 - 2.98 (m, 1H), 2.55 - 2.37 (m, 2H), 2.27–2.16 (m, 2H), 1.96–1.86 (m, 3H), 1.71 (s, 3H), 1.64 (t, $J$ = 11 .4 Hz, 2H), 1.59 - 1.20 (m, 16H), 1.08 (s, 3H), 1.03 (s, 3H), 1.00 (s, 3H), 0.99 (s, 3H), 0.94 (s, 3H). $^{13}$C NMR ($CDCl_3$): $\delta$ 218.2, 181.8, 150.3, 109.8, 56.3, 54.9, 49.8, 49.1, 47.3, 46.9, 42.4, 0.6, 38.5, 36.9, 34.1, 26.6, 20.9, 19.3, 15.9, 15.8, 14.6. LCMS (ESI) *m/z* calcd for $C_{30}H_{46}O_3$ $[M+H]^+$: 455.34. found: 455.33.

**SBI-0640599**[1]

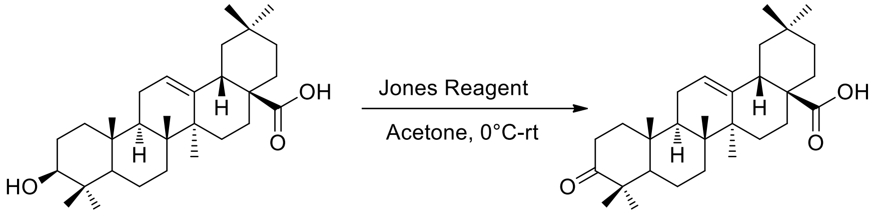


Synthesized according to **general method A** using Oleanolic acid  (5 g) and Jones reagent. White solid (4.7 g, 94%).$^{1}$H NMR ($CDCl_3$): $\delta$ 5.28 (s, 1H), 2.82 (dd, $J$ = 4.0 Hz, 13.7 Hz, 1H), 2.59–2.46 (m, 1H), 2.42–2.30 (m, 1H), 2.02–1.82 (m, 4H), 1.80–1.53 (m, 6H), 1.50–1.26 (m, 7H), 1.24–1.09 (overlapping singlet and multiplet, 6H), 1.08 (s, 3H), 1.03 (s, 3H), 1.00 (s, 3H), 0.90 (s, 3H), 0.88 (s, 3H), 0.78 (s, 3H). $^{13}$C NMR ($CDCl_3$): $\delta$ 217.8, 183.9, 143.6, 122.2, 55.3, 47.4, 46.8, 46.5, 45.8, 41.7, 40.9, 39.2, 39.1, 36.8, 34.1, 33.8, 33.0, 32.4, 32.1, 30.6, 27.6, 26.4, 25.8, 23.5, 23.4, 22.8, 21.4, 19.5, 16.9, 14.9. LCMS (ESI) *m/z* calcd for $C_{30}H_{46}O_3$ $[M+H]^+$: 455.34. found: 455.33.

**SBI-0640600**[1]

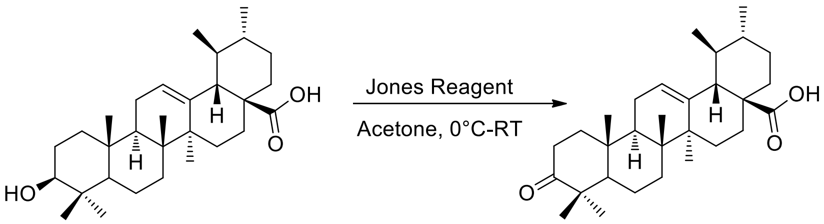


Synthesized according to **general method A** using Ursolic acid and Jones reagent. White powder (0.970 g, 97%). $^{1}$H NMR($CDCl_3$): $\delta$  5.24 (s, 1H), 2.57-2.48 (m, 1H), 2.41-2.35 (m, 1H), 2.18 (d, $J$ = 11.5 Hz, 1H), 2.22-1.24 (m, 20H), 1.06 (s, 6H), 1.05 (s, 3H), 1.02 (s, 3H), 0.96 (d, $J$ = 4.0  Hz, 3H), 0.84 (d, $J$ = 6.0 Hz, 3H), 0.79 (s, 3H). $^{13}$C NMR ($CDCl_3$): $\delta$ 217.7, 184.1, 138.5, 125.9, 55.6, 52.9, 48.4, 47.8, 42.7, 40.6, 39.8,

39.7, 39.4, 39.2, 37.1, 33.7, 31.0, 28.4, 26.9, 24.5, 23.8, 21.8, 21.6, 19.9, 19.6, 17.3, 15.9, 15.6. LCMS (ESI) *m/z* calcd for $C_{30}H_{46}O_3$ $[M+H]^+$: 455.34. found: 455.33.

**SBI-0061739**[2]

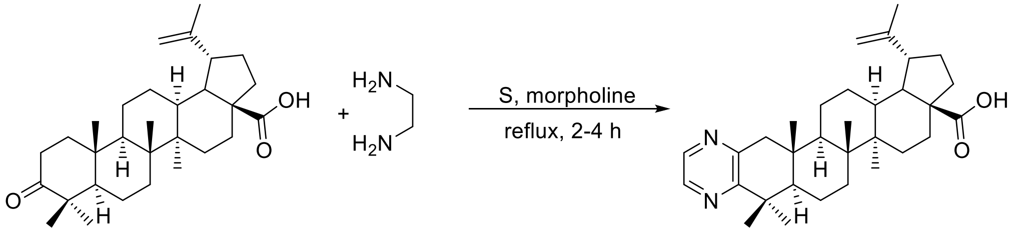


**General Method B**: To a solution of **SBI-0640601** (0.455 g, 1 mmol) in morpholine (5 mL) was added sulfur (0.320 g, 10 mmol) and ethylenediamine (0.33 mL, 10 mmol) at 23 °C. The resulting mixture was heated under reflux for 4 h. After cooling, the reaction mixture was poured into water and extracted with DCM (3 x 20 mL). The organic layer was washed with $H_2O$, diluted aqueous HCl, water, saturated aqueous $NaHCO_3$, and brine, dried over anhydrous sodium sulfate, and vacuum-concentrated. The crude product was purified by column chromatography (silica gel; EtOAc/petroleum ether) to yield the title compound as a white solid (0.294 g, 60%). $^1$H NMR ($CDCl_3$): $\delta$ 8.37 (d, $J$ = 2.1 Hz, 1H), 8.24 (d, $J$ = 2.2 Hz, 1H), 4.70 (s, 1H), 4.57 (s, 1H), 3.00−2.96 (m, 2H), 2.41−2.37 (m, 1H), 2.26−2.20 (m, 2H), 1.98−1.90 (m, 2H), 1.72−1.69 (m, 1H), 1.65 (s, 3H), 1.55−1.33 (m, 12H) 1.23 (s, 3H), 1.20 (s, 3H), 1.19−1.00 (m, 3H), 0.96 (s, 3H), 0.95 (s, 3H), 0.74 (s, 3H). $^{13}$C NMR ($CDCl_3$): $\delta$ 181.6, 159.7, 150.7, 150.3, 142.3, 142.2, 109.7, 56.4, 53.0, 49.2, 48.7,48.4, 46.9, 42.5, 40.5, 39.4, 38.4, 37.0, 36.7, 33.3, 32.1, 31.4, 30.6, 29.7,25.4, 23.9, 21.3, 20.0, 19.4, 16.0, 15.6, 14.6. HRMS (ESI) *m/z* calcd for $C_{32}H_{46}N_2O_2$ $[M+H]^+$: 491.3578. found: 491.3629.

**SBI-0069275**[3]

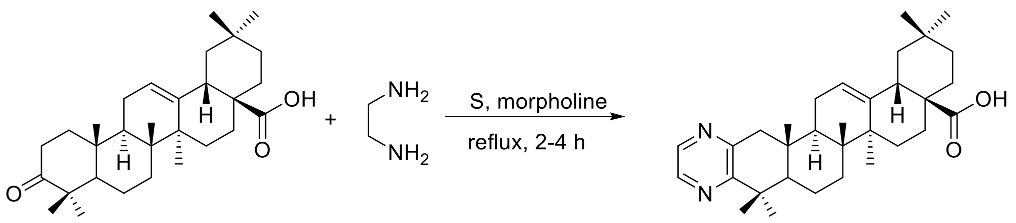


Synthesized according to **general method B** using **SBI-0640599** (0.455 g, 1 mmol), ethylenediamine (0.33 mL, 5 mmol) and sulfur (0.320 g, 10 mmol). White powder (0.332 g, 67%). $^1$H NMR ($CDCl_3$): $\delta$ 8.44 (s,

1H), 8.28 (s, 1H), 5.38 (t, $J$ = 3.0 Hz, 1H), 3.00 (d, $J$ = 15.0 Hz, 1H), 2.87 (dd, $J$ = 3.0 Hz, 15.0 Hz, 1H), 2.52 (d, $J$ = 15.0 Hz, 1H), 2.07-2.00 (m, 3H), 1.79-1.33 (m, 16H), 1.31 (s, 3H), 1.28 (s, 3H), 1.19 (s, 3H), 0.94 (s, 3H), 0.93 (s, 3H), 0.92 (s, 3H), 0.86 (s, 3H). $^{13}$C NMR ($CDCl_3$): $\delta$ 183.7, 159.8, 150.4, 143.5, 142.4, 141.2, 122.4, 53.1, 48.1, 46.7, 45.9, 41.9, 41.3, 39.3, 36.7, 33.2, 31.7, 30.8, 27.8, 25.9, 24.3, 23.7, 20.2, 16.9, 15.6. HRMS (ESI) $m/z$ calcd for $C_{32}H_{46}N_2O_2$ $[M+H]^+$: 491.3561. found: 491.3632.

**SBI-0069273**

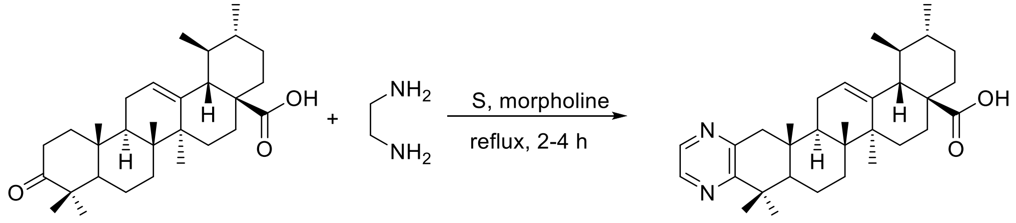


Synthesized according to **general method** B using **SBI-0640600** (0.455g, 1 mmol), ethylenediamine (0.33mL, 5 mmol) and sulfur (0.320 g, 10 mmol). White powder (0.300 g, 61%). $^1$H NMR ($CDCl_3$): $\delta$ 8.43 (s, 1H), 8.29 (s, 1H), 5.33 (t, $J$ = 3.3 Hz, 1H), 3.03 (d, $J$ = 16.5 Hz, 1H), 2.50 (d, $J$ = 16.0 Hz, 1H), 2.25 (d, $J$ = 11.0 Hz, 1H), 2.07-1.44 (overlapping singlet and multiplets, 15H), 1.42 (s, 3H), 1.38 (s, 3H), 1.15 (s, 3H), 1.04-0.96 (m, 3H), 0.95 (d, $J$ = 6.8 Hz, 3H), 0.94 (s, 3H), 0.91 (d, $J$ = 7.5 Hz, 3H), 0.89 (s, 3H). $^{13}$C NMR ($CDCl_3$): $\delta$ 183.4, 159.7, 150.5, 142.3, 141.3, 137.9, 125.6, 52.9, 52.8, 48.1, 48.0, 42.1, 39.3, 38.8, 31.3, 31.5, 30.5, 24.1, 23.4, 21.2, 17.0, 16.8, 15.6. HRMS (ESI) $m/z$ calcd for $C_{32}H_{46}N_2O_2$ $[M+H]^+$: 491.3557. found: 491.3629.

**SBI-0069274**

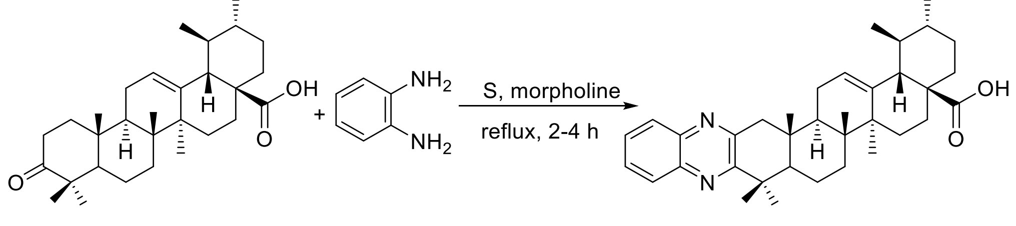


Synthesized according to **general method B** using **SBI-0640600** (0.455 g, 1 mmol), o-phenylenediamine (0.540 g, 5 mmol) and sulfur (0.320 g, 10 mmol). White powder (0.359 g, 66%). $^1$H NMR ($CDCl_3$): $\delta$ 8.02−7.96 (m, 2H), 7.72−7.59 (m, 2H), 5.35 (t, $J$ = 3.4 Hz, 1H), 3.28 (d, $J$ = 16.5 Hz, 1H), 2.68 (d, $J$ = 16.5

Hz, 1H), 2.26 (d, $J$ = 11.2 Hz, 1H), 2.16−1.48 (m, 15H), 1.42 (s, 3H), 1.38 (s, 3H), 1.15 (s, 3H), 0.95 (d, $J$ = 6.8 Hz, 3H), 0.94 (s, 3H), 0.92-0.90 (overlapping doublet and multiplets, 6H), 0.89 (s, 3H). $^{13}C$ NMR ($CDCl_3$) $\delta$: 183.1, 161.1, 151.9, 142.1, 137.9., 129.3, 128.8, 127.3, 125.6, 52.4, 52.7, 48.9, 48.0, 45.4, 42.2, 40.4, 39.4, 39.1, 38.8, 36.8, 32.3, 30.6, 29.7, 28.0, 25.3, 24.1, 23.5, 23.3, 21.1, 20.2, 17.0, 16.8, 15.8. HRMS (ESI) *m/z* calcd for $C_{36}H_{48}N_2O_2$ $[M+H]^+$: 541.3713. found: 540.3785.

**SBI-0069272**[2]

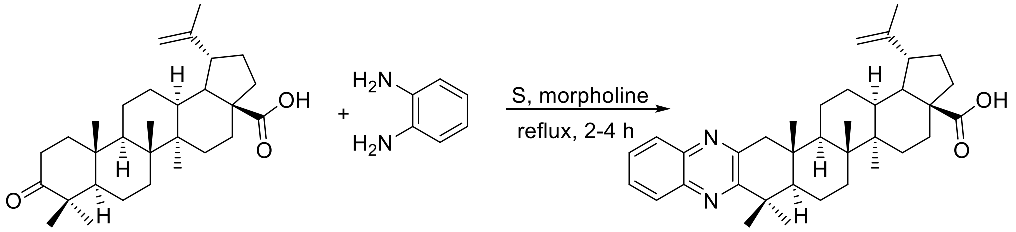


Synthesized according to **general method B** using **SBI-0640601** (0.455 g, 1 mmol), o-phenylenediamine (0.540 g, 5 mmol) and sulfur (0.320 g, 10 mmol). White powder (0.313 g, 58%). $^1H$ NMR ($CDCl_3$): $\delta$ 8.02-7.96 (m, 2H), 7.66-7.64 (m, 2H), 4.78 (d, $J$ = 1.8 Hz, 1H), 4.66 (s, 1H), 3.32 (d, $J$ = 16.2 Hz, 1H), 3.08-2.94 (m, 1H), 2.59 (d, $J$ = 16.5 Hz, 1H), 2.33-2.26 (m, 2H), 2.03-1.97 (m, 2H), 1.78 (d, $J$ = 13.7 Hz, 1H), 1.73 (s, 3H), 1.70-1.48 (m, 16H), 1.44 (s, 3H), 1.42 (s, 3H), 1.06 (s, 3H), 1.04 (s, 3H), 0.84 (s, 3H). $^{13}C$ NMR ($CDCl_3$): $\delta$ 181.7, 161.2, 152.3, 150.3, 142.2, 140.8, 128.8, 128.6, 109.8, 56.5, 53.5, 49.7, 49.3, 48.6, 46.9, 42.6, 40.7, 40.5, 38.6, 37.2, 33.4, 32.2, 30.7, 29.8, 25.6, 25.2, 21.5, 20.4, 19.6, 16.3, 15.7, 14.8. HRMS (ESI) *m/z* calcd for $C_{36}H_{48}N_2O_2$ $[M+H]^+$: 541.3722. found: 541.3782.

**SBI-0069276**

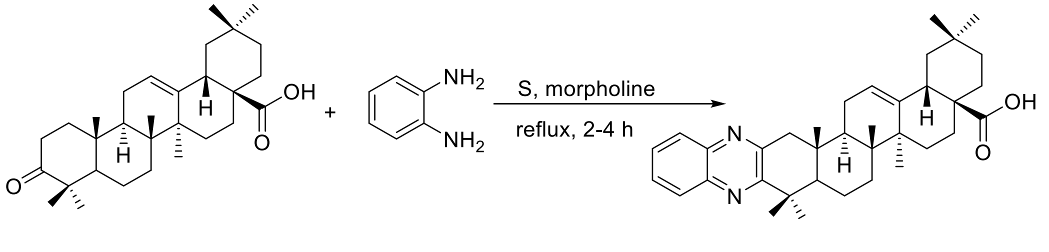


Synthesized according to **general method B** using **SBI-0640599** (0.455 g, 1 mmol), o-phenylenediamine (0.540 g, 5 mmol) and sulfur (0.320 g, 10 mmol). White powder (0.368 g, 68%). $^1H$ NMR ($CDCl_3$) $\delta$: 8.03–7.96 (m, 2H), 7.68–7.64 (m, 2H), 5.39 (t, $J$ = 2.8 Hz, 1H), 3.26 (d, $J$ = 15.0 Hz, 1H), 2.88 (dd, $J$ = 3.0 Hz,

15.0 Hz, 1H), 2.66 (d, $J$ =15.0 Hz, 1H), 2.11-1.59 (m, 5H), 1.39 (s, 3H), 1.38 (s, 3H), 1.19 (s, 3H), 1.24-0.96 (m, 14H), 0.94 (s, 3H), 0.93 (s, 3H), 0.92 (s, 3H), 0.84 (s, 3H). $^{13}$C NMR($CDCl_3$) $\delta$: 184.0, 161.2, 152.0, 143.6, 142.3, 140.7, 129.0, 128.9, 128.7, 128.0, 122.7, 53.6, 49.3, 46.7, 45.7, 42.0, 41.2, 40.5, 39.4, 37.0,34.0, 33.2, 32.4, 30.8, 27.8, 25.9, 25.5, 23.7, 20.5, 16.9, 15.7. HRMS (ESI) *m/z* calcd for $C_{36}H_{48}N_2O_2$ $[M+H]^+$: 541.3716. found: 541.3787.

**SBI-0640716**[4]

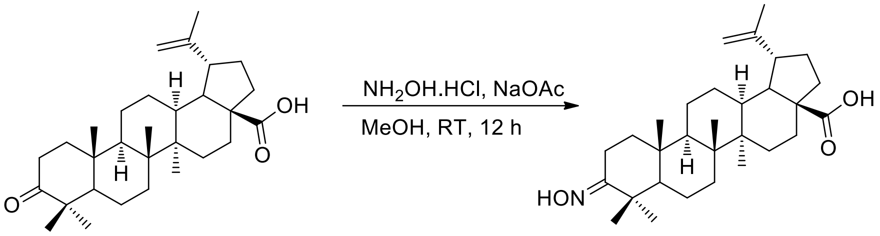


**General Method C**: $NH_2OH.HCl$ (0.060 g, 0.88 mmol) was added to a solution of **SBI-0646601** (0.200 g, 0.44 mmol) in dry methanol (5 mL) and sodium acetate (0.072 g, 0.88 mmol). The reaction mixture was stirred for 12 h at room temperature under nitrogen atmosphere. After addition of ice water (100 mL), the precipitated solid was filtered and washed with water (twice) to give a white solid (0. 200 g, 97%). $^1$H NMR ($CDCl_3$): $\delta$ 7.56 (s, 1H), 4.72 (s, 1H), 4.61 (s, 1H), 3.02-2.89 (m, 3H), 2.54-2.47 (m, 1H), 1.97-1.73 (m, 4H), 1.79 (s, 3H), 1.63-1.12 (m, 12H), 1.38 (s, 3H), 1.14 (s, 3H), 1.10-1.08 (m, 4H), 1.07 (s, 3H), 1.05 (s, 3H), 0.89 (s, 3H). $^{13}$C NMR ($CDCl_3$): $\delta$ 179.3, 164.7, 151.6, 110.3, 57.0, 56.3, 51.0, 50.0, 48.1, 43.2, 41.4, 40.6, 39.5, 39.0, 38.0, 37.8, 34.7, 33.2, 31.5, 30.6, 28.3, 26.4, 23.8, 21.8, 19.8, 19.7, 17.8, 16.6, 16.2, 15.1. HRMS (ESI) *m/z* calcd for $C_{30}H_{47}NO_3$ $[M+H]^+$: 470.3551. found: 470.3625.

**SBI-0640718**[5]

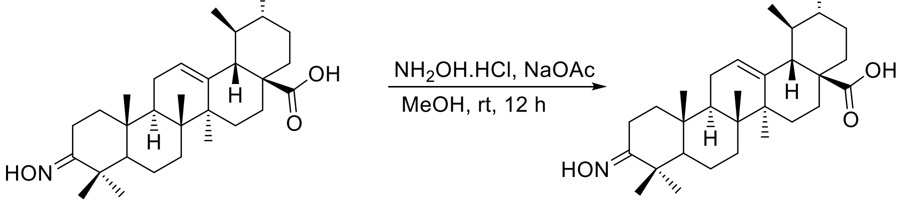


Prepared according to **general method C** using **SBI-0640600** (2.00 g, 4.4 mmol), $NH_2OH$, HCl (0.60 g, 8.8 mmol) and sodium acetate (0.72 g, 8.8 mmol). The reaction mixture was stirred for 12 h at room temperature

under nitrogen atmosphere. After addition of ice water, the precipitated solid was filtered and washed with water (twice) to give a white solid (2.00 g, 97%). $^1$H NMR ($CDCl_3$): $\delta$ 5.26 (t, $J$ = 3.3 Hz, 1H), 3.02 (d, $J$ = 15.0 Hz, 1H), 2.22-2.19 (m, 2H), 2.08-1.88 (m, 4H), 1.78-1.65 (m, 4H), 1.55-1.46 (m, 4H), 1.40-1.24 (m, 6H), 1.13 (s, 3H), 1.07 (s, 3H), 1.06 (s, 3H), 1.04 (s, 3H), 1.02 (s, 3H), 0.94 (d, $J$ = 6.0 Hz, 3H), 0.86 (d, $J$ = 6.4 Hz, 3H), 0.81 (s, 3H). $^{13}$C NMR ($CDCl_3$): $\delta$ 183.2, 167.3, 138.0, 125.6, 55.5, 52.7, 47.8, 46.9, 42.0, 40.2, 39.5, 39.0, 38.8, 38.3, 36.9, 36.7, 32.6, 30.6, 27.9, 27.4, 24.1, 23.5, 23.4, 21.6, 19.0, 17.6, 16.9. HRMS (ESI) *m/z* calcd for $C_{30}H_{47}NO_3$ $[M+H]^+$: 470.3551. found: 470.3625.

**SBI-0640720**[5a]

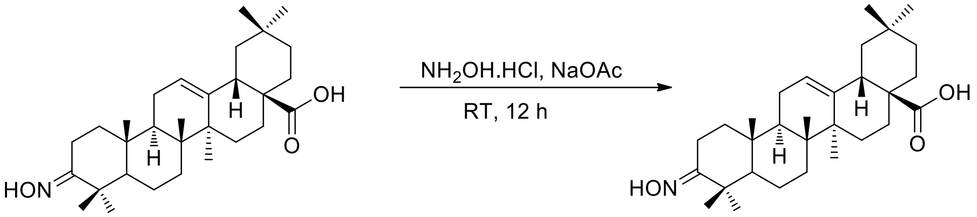


Synthesized according to **general method C** using **SBI-0640599** (1 g, 2.2 mmol), $NH_2OH.HCl$ (0.303 g, 4.4 mmol) and sodium acetate (0.360 g, 4.4 mmol). White solid (2 g, quantitative yield). $^1$H NMR ($CDCl_3$): $\delta$ 5.27 (s, 1H), 3.03 (d, $J$ = 15.6 Hz, 1H), 2.82 (d, $J$ = 13.3 Hz, 1H), 2.13-2.05 (m, 2H), 1.97-1.89 (m, 6H), 1.51-1.19 (m, 12H), 1.20 (s, 3H), 1.12 (s, 3H), 1.02 (s, 3H), 1.01 (s, 3H), 0.92 (s, 3H), 0.89 (s, 3H), 0.83 (s, 3H). $^{13}$C NMR ($CDCl_3$): $\delta$ 178.7, 163.6, 142.9, 120.8, 54.6, 45.9, 44.9, 44.8, 40.6, 40.0, 37.2, 35.8, 32.7, 32.1, 31.3, 29.6, 26.5, 24.7, 22.5, 22.2, 21.9, 17.9, 15.9, 15.7, 13.7. HRMS (ESI) *m/z* calcd for $C_{30}H_{47}NO_3$ $[M+H]^+$: 470.3547. found: 470.3625.

**SBI-0640717**[5]

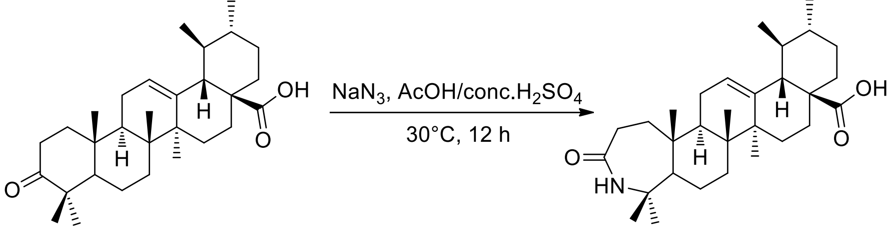


To a solution of **SBI-0646600** (1 g, 3.2 mmol) in glacial acetic acid (30 mL) was added concentrated $H_2SO_4$ (0.4 mL) and heated at 65 ºC-70 ºC. To this sodium azide (0.5 g, 7.7 mmol) was added in parts during 30

min. with occasional stirring. After the addition of sodium azide, the reaction mixture was kept at 30 ºC for 12 h. Poured into ice cooled sodium carbonate solution and extracted with ether (3 x 100 mL). Organic layer washed with water, brine and dried over anhydrous $Na_2SO_4$. Removal of the solvent followed by silica gel column chromatography using chloroform yielded the title compound as a white solid (0.850 g, 57%). $^1$H NMR ($CDCl_3$): $\delta$ 6.52 (s, 1H), 5.26 (s, 1H), 2.58-2.43 (m, 2H), 2.21 (d, $J$ = 11.4 Hz, 1H), 2.02-1.33 (m, 20H), 1.29 (s, 3H), 1.25 (s, 3H), 1.11 (s, 3H), 1.07 (s, 3H), 0.96 (d, $J$ = 6.1 Hz, 3H), 0.84 (d, $J$ = 6.1 Hz, 3H), 0.79 (s, 3H). $^{13}$C NMR ($CDCl_3$): $\delta$ 182.7, 177.7, 137.9, 125.5, 56.5, 55.1, 52.5, 47.8, 47.3, 42.2, 40.7, 39.6, 39.0, 38.8, 37.5, 36.6, 34.0, 32.6, 31.5, 30.6, 27.9, 26.3, 23.9, 23.7, 23.3, 21.8, 21.2, 17.1, 16.9, 16.7. HRMS (ESI) *m/z* calcd for $C_{30}H_{47}NO_3$ $[M+H]^+$: 470.3553. found: 470.3627.

**SBI-0640719**[5a]

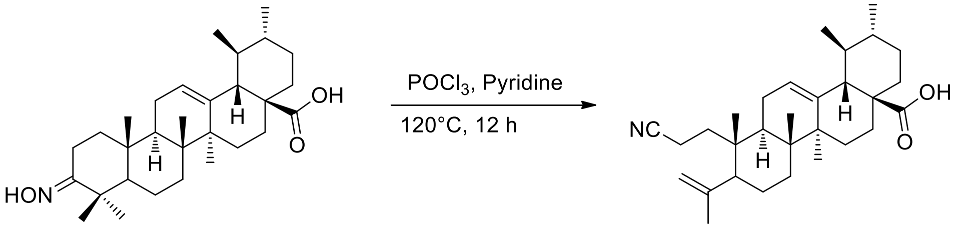


To a solution of **SBI-0640718** (0.900 g, 1.92 mmol) in pyridine (30 mL) was added $POCl_3$ (2 mL). The resulting mixture was heated at 120 ºC for 12 h. Cooled to room temperature and removed the solvent under reduced pressure. Poured into ice water and extracted with ether (3 x 100 mL). Organic layer washed with water, diluted HCl, brine and dried over anhydrous $Na_2SO_4$. Removal of the solvent followed by silica gel column chromatography using hexanes:ethyl acetate yielded the title compound as a white solid (0.690 g, 79%). $^1$H NMR ($CDCl_3$): $\delta$ 5.27 (s, 1H), 4.89 (s, 1H), 4.64 (s, 1H), 2.35-2.19 (m, 3H), 2.10-1.83 (m, 6H), 1.75 (s, 3H), 1.69-1.25 (m, 14H), 1.15-1.02 (overlapping singlet and multiplets, 6H), 0.97 (d, $J$ = 6.1 Hz, 3H), 0.88 (d, $J$ = 6.0 Hz, 3H), 0.83 (s, 3H). $^{13}$C NMR ($CDCl_3$): $\delta$ 183.4, 146.7, 138.2, 125.1, 120.2, 114.2, 52.6, 50.6, 47.9, 42.3, 39.4, 39.2, 39.0, 38.7, 37.7, 34.3, 31.5, 30.6, 27.9, 24.0, 23.9, 23.6, 23.4, 22.9, 21.1, 19.2, 17.2, 17.0, 11.6. HRMS (ESI) *m/z* calcd for $C_{30}H_{45}NO_2$ $[M+H]^+$: 452.3429. found: 452.3524.

**SBI-0069325**[6]

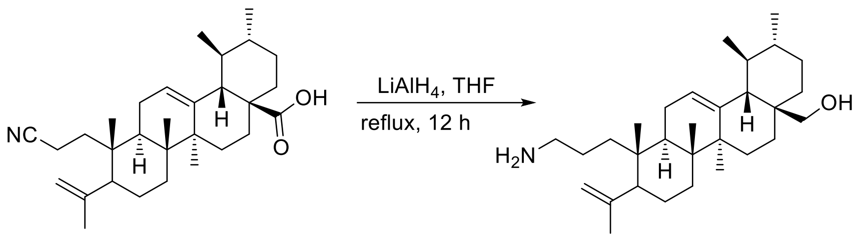


To a cooled solution of **SBI-0640719** (0.451 g, 1 mmol) in dry THF (20 mL) was added $LiAlH_4$ solution in drop-wise (2M $LiAlH_4$ solution in THF, 2 mL) and the resulting mixture was refluxed for 12 h. The reaction mixture was then cooled to room temperature and then to 0 ºC. Excess reagent was quenched by the addition of 1N HCl: 3N NaOH: $H_2O$ (1:1:3). The precipitated white solid was filtered off. Filtrate washed with water, dried over anhydrous $Na_2SO_4$ and evaporated to give the crude product, which was purified by column chromatography (hexanes: ethyl acetate) to yield the corresponding product. White solid (0.380 g, 86%). $^1$H NMR ($CDCl_3$): $\delta$ 5.15 (t, $J$ = 4.6 Hz, 1H), 4.84 (s, 1H), 4.65 (s, 1H), 3.53 (d, $J$ = 11.0 Hz, 1H), 3.20 (d, $J$ = 11.0 Hz, 1H), 2.65-2.61 (m, 1H), 2.09-2.10 (m, 1H), 1.96-1.77 (m, 10H), 1.75 (s, 3H), 1.60-1.18 (m, 12H), 1.14 (s, 3H), 1.12-1.02 (m, 1H), 1.03 (s, 3H), 0.94 (d, $J$ = 6.0 Hz, 3H), 0.91 (s, 3H), 0.87-0.88 (m, 1H), 0.83 (d, $J$ = 6.0 Hz, 3H). $^{13}$C NMR ($CDCl_3$): $\delta$ 148.0, 138.7, 125.1, 113.1, 69.9, 54.1, 50.3, 42.8, 42.5, 39.6, 39.4, 39.0, 38.0, 37.7, 36.6, 35.2, 31.5, 30.6, 29.8, 27.0, 25.9, 24.4, 23.6, 23.4, 23.3, 23.2, 21.3, 20.2, 17.5, 16.9. HRMS (ESI) $m/z$ calcd for $C_{30}H_{51}NO$ $[M+H]^+$: 442.3967. found: 442.4040.

**SBI-0069327**

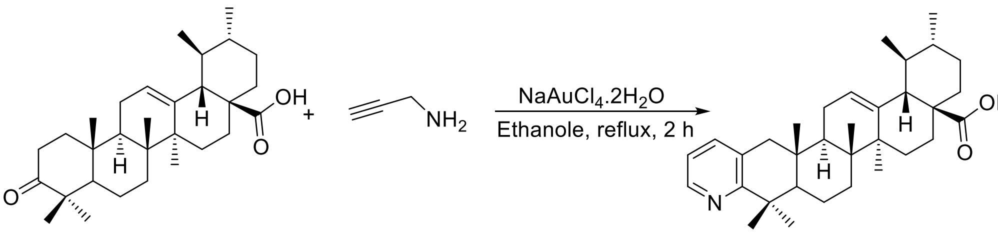


**SBI-0640600** (0.455 g, 1 mmol), propargylamine (0.110 g, 2 mmol) and $NaAuCl_4.2H_2O$ (12 mg, 0.03 mmol) were taken in ethanol (5 mL) and refluxed for 2-4 h under nitrogen atmosphere. Cooled, filtered off the catalyst and removed the solvent under reduced pressure. Crude material was purified by column chromatography using hexane:ethylacetate. White solid (0.300 g, 61%). $^1$H NMR ($CDCl_3$): $\delta$ 9.02 (d, $J$ = 5.0

Hz, 1H), 7.92 (d, $J$ = 7.8 Hz, 1H), 7.59-7.55 (m, 1H), 5.32 (t, $J$ = 3.2 Hz, 1H), 2.93 (d, $J$ = 16.5 Hz, 1H), 2.56 (d, $J$ = 16.0 Hz, 1H), 2.23 (d, $J$ = 11.5 Hz, 1H), 2.08-1.88 (m, 4H), 1.78-1.57 (m, 12H), 1.55 (s, 3H), 1.50-1.43 (m, 1H), 1.48 (s, 3H),1.37-1.32 (m, 2H), 1.18-1.00 (m, 1H), 1.12 (s, 3H), 1.09-1.00 (m, 1H), 0.96 (d, $J$ = 6.1 Hz, 3H), 0.88 (d, $J$ = 6.1 Hz, 3H), 0.87 (s, 3H). $^{13}$C NMR ($CDCl_3$): $\delta$ 182.7, 160.1, 145.5, 141.9, 138.4, 134.6, 124.8, 123.3, 52.9, 52.7, 47.9, 45.6, 44.8, 42.3, 39.3, 39.1, 38.8, 36.5, 35.9, 32.1, 30.5, 30.4, 27.8, 24.0, 23.4, 23.3, 21.1, 19.5, 16.9, 16.6, 15.3. HRMS (ESI) *m/z* calcd for $C_{33}H_{47}NO_2$ $[M+H]^+$: 490.3606. found: 490.3677.

**SBI-0069328**

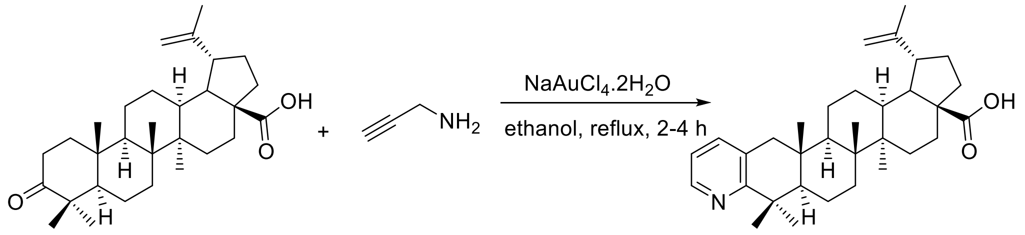


**SBI-0640601** (0.200 g, 0.44 mmol), propargylamine (0.048 g, 0.88 mmol) and $NaAuCl_4.2H_2O$ ( 5.2 mg, 0.013 mmol) were taken in ethanol (2 mL) and refluxed for 2-4 h under nitrogen atmosphere. Cooled, filtered off the catalyst and removed the solvent under reduced pressure. Crude material was purified by column chromatography using hexane:ethylacetate. White solid (0.123 g, 56%). $^1$H NMR ($CDCl_3$): $\delta$ 8.48 (d, $J$ = 4.6 Hz, 1H), 7.27 (d, $J$ = 7.3 Hz, 1H), 7.04-7.01 (m, 1H), 4.75 (s, 1H), 4.62 (s, 1H), 3.20-3.07 (m, 1H), 2.74 (d, $J$ = 15.5 Hz, 1H), 2.36-2.26 (m, 3H), 2.03-1.97 (m, 2H), 1.79-1.34 (overlapping singlets and multiplets, 16H), 1.71 (s, 3H), 1.32 (s, 3H), 1.27 (s, 3H), 1.02 (s, 3H), 1.00 (s, 3H), 0.78 (s, 3H). $^{13}$C NMR ($CDCl_3$): $\delta$ 180.6, 163.3, 150.7, 146.6, 138.3, 130.1, 120.9, 109.6, 56.3, 53.5, 49.2, 48.8, 46.9, 45.8, 42.5, 40.5, 39.4, 38.4, 37.0, 36.2, 31.3, 30.6, 29.7, 25.6, 23.9, 21.5, 20.2, 19.4, 15.7, 14.6. HRMS (ESI) *m/z* calcd for $C_{33}H_{47}NO_2$ $[M+H]^+$: 490.3602. found: 490.3676.

**References**


1. Huang L, Luo H, Yang X, Chen L, Zhang J, Wang D and Hao X. Enhancement of anti-bacterial and anti-tumor activities of pentacyclic triterpenes by introducing exocyclic α,β-unsaturated ketone moiety in ring A. Medicinal Chemistry Research. 2014; 23(11):4631-4641.
2. Urban M, Sarek J, Kvasnica M, Tislerova I and Hajduch M. Triterpenoid pyrazines and benzopyrazines with cytotoxic activity. J Nat Prod. 2007; 70(4):526-532.

3. Li JF, Zhao Y, Cai MM, Li XF and Li JX. Synthesis and evaluation of a novel series of heterocyclic oleanolic acid derivatives with anti-osteoclast formation activity. Eur J Med Chem. 2009; 44(7):2796-2806.
4. Genet C, Strehle A, Schmidt C, Boudjelal G, Lobstein A, Schoonjans K, Souchet M, Auwerx J, Saladin R and Wagner A. Structure-activity relationship study of betulinic acid, a novel and selective TGR5 agonist, and its synthetic derivatives: potential impact in diabetes. J Med Chem. 2010; 53(1):178-190.
5. Sundraramaiah T, Ramraj S, Rao L and Bai V. Synthesis of A-Aza triterpenes-1: A-Aza triterpenes from methyl oleanoate, methyl betulonate and lupenone. J Indian Chem Soc 1976:664-665.
6. Gnoatto SC, Dassonville-Klimpt A, Da Nascimento S, Galera P, Boumediene K, Gosmann G, Sonnet P and Moslemi S. Evaluation of ursolic acid isolated from Ilex paraguariensis and derivatives on aromatase inhibition. Eur J Med Chem. 2008; 43(9):1865-1877.
7. Wrzeciono U, Turowska W and Kusnierkiewicz G. Nitrogen-containing triterpenes. IV. 3,4-Seco- and A-homooleane derivatives. Rocz Chem. 1971; 45(7/8):1233-1240.

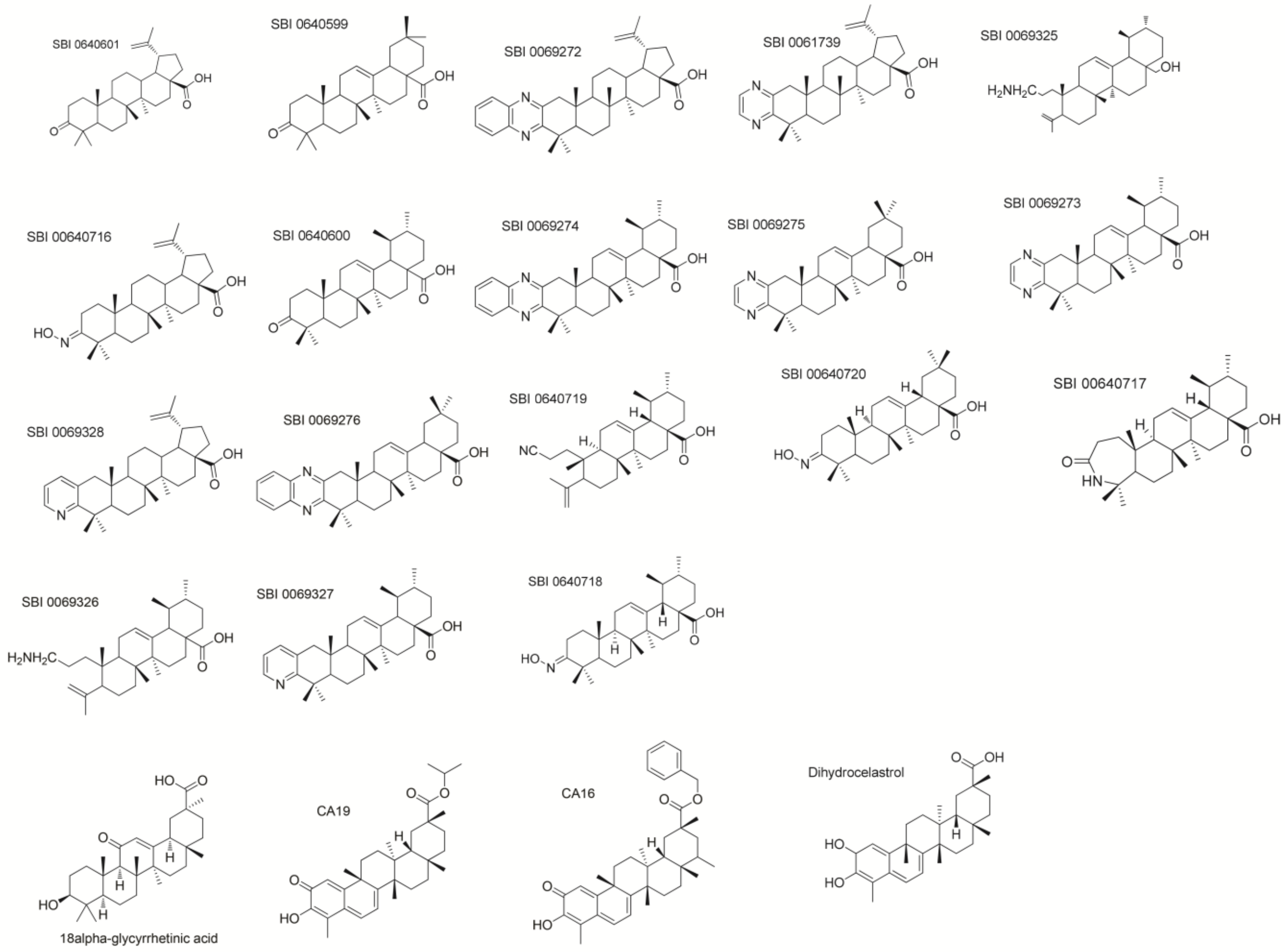


**Supplementary Figure 1.** Structures of SBI compounds and other triterpenoid analogs (see Figure 1A for structure of celastrol).

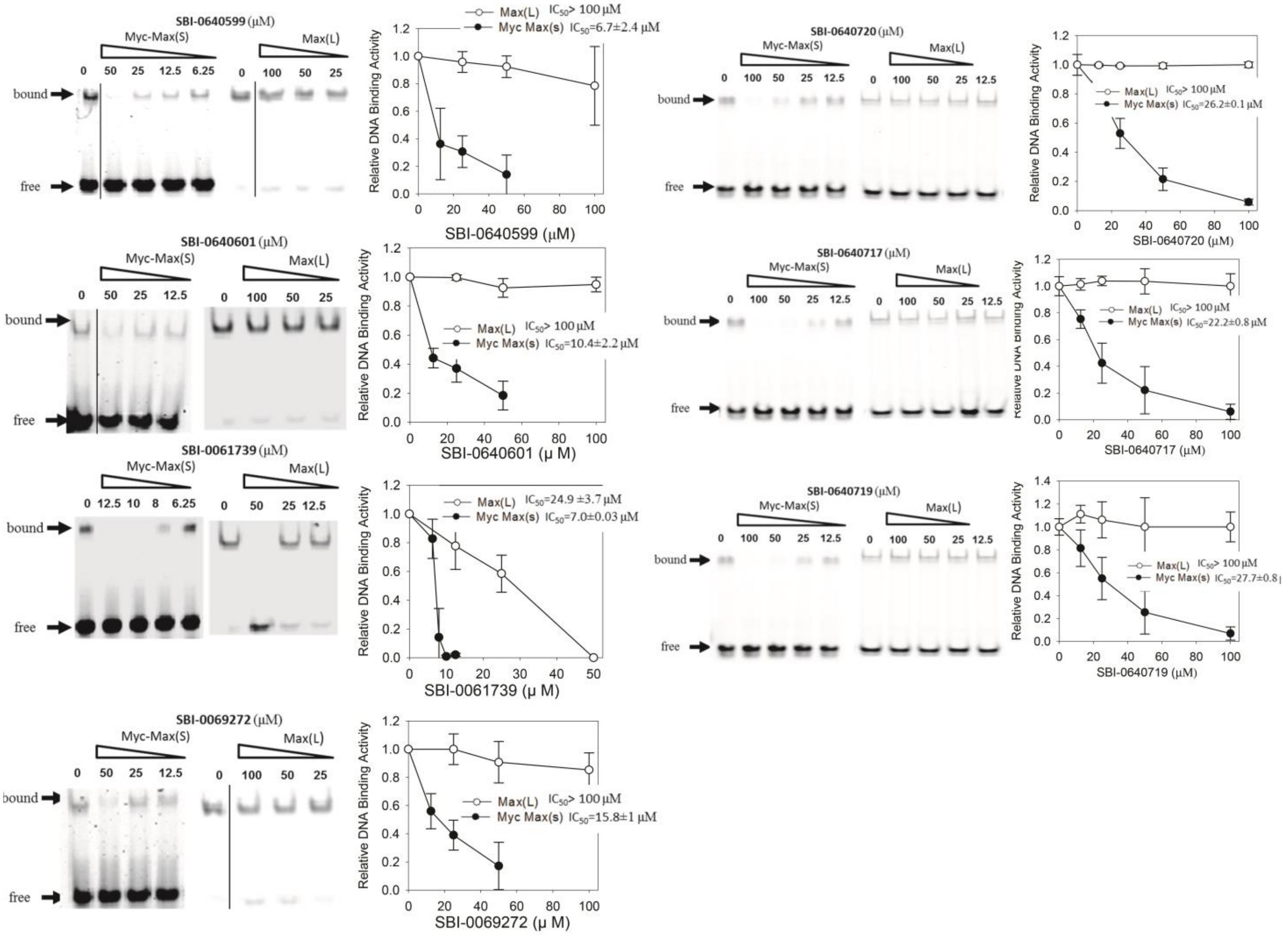


**Supplementary Figure 2.** EMSAs of celastrol analogs. After initial screens of each of the analogs shown in Supplementary Figure 1 showed some to be active at 100 μM concentrations, EMSAs were repeated as described in Figure 1.

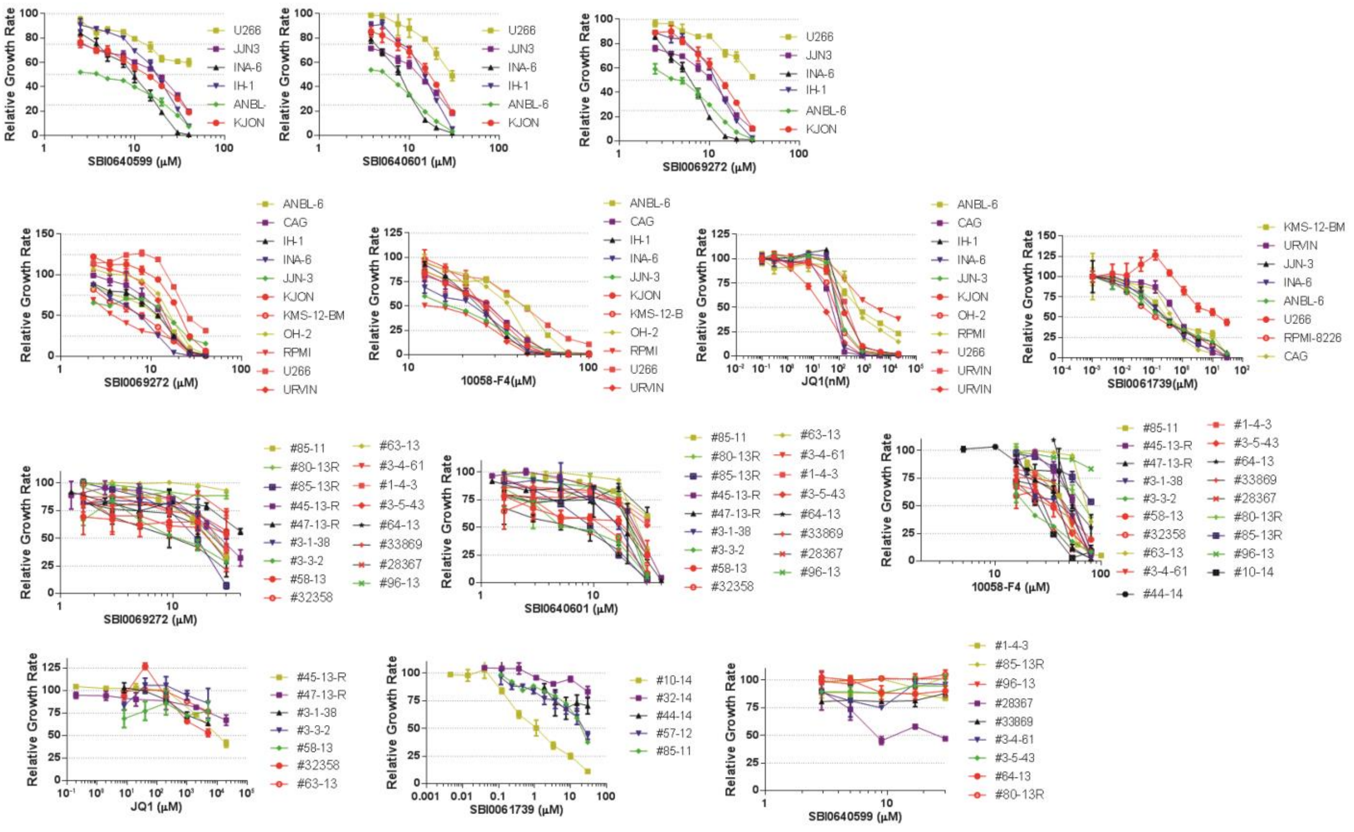


**Supplementary Figure 3.** Inhibition of myeloma cell lines and primary myeloma xenografts. Individual multi-point dose-response curves used to generate the histograms depicted in Figure 6 are shown.

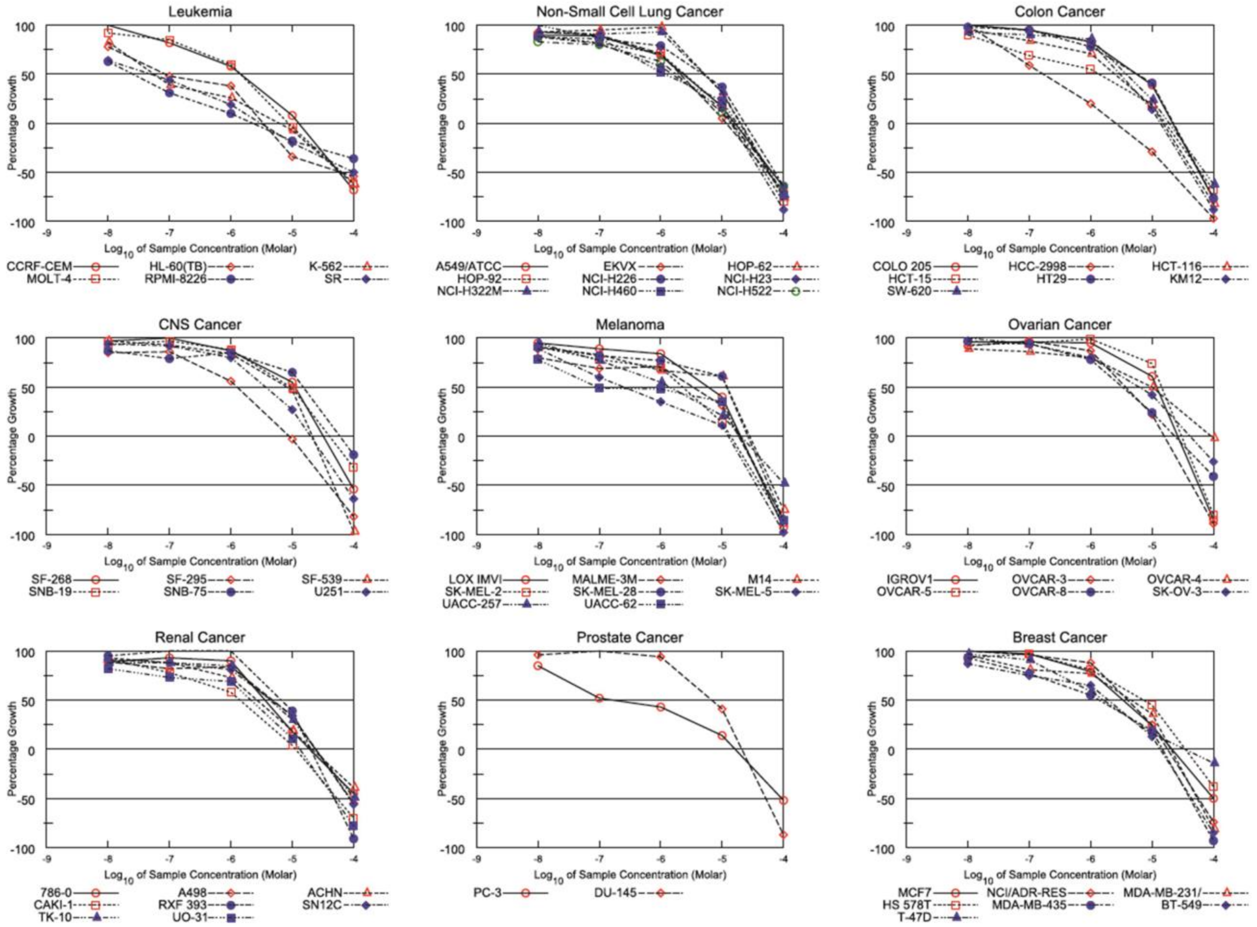


**Supplementary Figure 4.** Multi-point dose-response curves for celastrol analog SBI-0061739 performed against the National Cancer Institute NCI-60 cell line collection.
